# Experimental Traumatic Brain Injury Induces Chronic Glutamatergic Dysfunction in Amygdala Circuitry Known to Regulate Anxiety-Like Behavior

**DOI:** 10.3389/fnins.2019.01434

**Published:** 2020-01-21

**Authors:** Joshua A. Beitchman, Daniel R. Griffiths, Yerin Hur, Sarah B. Ogle, Caitlin E. Bromberg, Helena W. Morrison, Jonathan Lifshitz, P. David Adelson, Theresa Currier Thomas

**Affiliations:** ^1^Barrow Neurological Institute at Phoenix Children’s Hospital, Phoenix, AZ, United States; ^2^Department of Child Health, University of Arizona College of Medicine-Phoenix, Phoenix, AZ, United States; ^3^College of Graduate Studies, Midwestern University, Glendale, AZ, United States; ^4^Banner University Medical Center, Phoenix, AZ, United States; ^5^College of Nursing, University of Arizona, Tucson, AZ, United States; ^6^Phoenix VA Health Care System, Phoenix, AZ, United States

**Keywords:** diffuse traumatic brain injury, amperometry, glutamate neurotransmission, amygdala, chronic

## Abstract

Up to 50% of traumatic brain injury (TBI) survivors demonstrate persisting and late-onset anxiety disorders indicative of limbic system dysregulation, yet the pathophysiology underlying the symptoms is unclear. We hypothesize that the development of TBI-induced anxiety-like behavior in an experimental model of TBI is mediated by changes in glutamate neurotransmission within the amygdala. Adult, male Sprague-Dawley rats underwent midline fluid percussion injury or sham surgery. Anxiety-like behavior was assessed at 7 and 28 days post-injury (DPI) followed by assessment of real-time glutamate neurotransmission in the basolateral amygdala (BLA) and central nucleus of the amygdala (CeA) using glutamate-selective microelectrode arrays. The expression of anxiety-like behavior at 28 DPI coincided with decreased evoked glutamate release and slower glutamate clearance in the CeA, not BLA. Numerous factors contribute to the changes in glutamate neurotransmission over time. In two additional animal cohorts, protein levels of glutamatergic transporters (Glt-1 and GLAST) and presynaptic modulators of glutamate release (mGluR2, TrkB, BDNF, and glucocorticoid receptors) were quantified using automated capillary western techniques at 28 DPI. Astrocytosis and microglial activation have been shown to drive maladaptive glutamate signaling and were histologically assessed over 28 DPI. Alterations in glutamate neurotransmission could not be explained by changes in protein levels for glutamate transporters, mGluR2 receptors, astrocytosis, and microglial activation. Presynaptic modulators, BDNF and TrkB, were significantly decreased at 28 DPI in the amygdala. Dysfunction in presynaptic regulation of glutamate neurotransmission may contribute to anxiety-related behavior and serve as a therapeutic target to improve circuit function.

## HIGHLIGHTS

- A single diffuse brain injury induces anxiety-like behavior in an open field environment.
- Altered glutamate neurotransmission occurs following diffuse TBI in the CeA, but not in the BLA.
- The expression of affective symptoms coincides with altered glutamate neurotransmission in the CeA.
- Altered glutamate neurotransmission occurs in the absence of overt neuropathology and gliosis.
- BDNF and TrkB were significantly decreased at 28 DPI in the amygdala.

## Introduction

Affective disorders, including generalized anxiety disorder and post-traumatic stress disorder (PTSD), develop and persist in up to 50% of traumatic brain injury (TBI) survivors, however, few common biological mechanisms between TBI and non-TBI patients have been elucidated (Kessler et al., 2005; Scholten et al., 2016). The incidence of TBI continues to rise, with at least 2.5 million Americans reporting a TBI each year, costing the American health care system 76.5 billion dollars (Hyder et al., 2007; Coronado et al., 2012). Three-quarters of all TBIs are diffuse TBIs (dTBI) in which the signature pathology is multi-focal diffuse axonal injury with no overt pathology detected by CT or MRI (Lee and Newberg, 2005; Grossman et al., 2010; Liu et al., 2014). dTBI subsequently induces secondary sequelae that occur seconds to months following the initial injury, leading to the development of affective, cognitive, and somatic symptoms (McAllister, 1992; Masel and DeWitt, 2010). Despite clinical prevalence, the pathophysiology contributing to affective symptoms following dTBI is poorly understood, resulting in misdiagnosis and ineffective treatments (Van der Kolk, 2002; Collins et al., 2004; Hoge et al., 2014). Ultimately, ineffective treatment impacts a patient’s ability to return to work, daily function, and social interactions; severely impairing the quality of life for both the patient and caregivers (Trahan et al., 2001; Guerin et al., 2006; van der Horn et al., 2013).

Clinical and preclinical data demonstrate that dTBI causes profound lasting changes in the amygdala. In Veterans, early amygdala fMRI reactivity post-injury is predictive of the development of PTSD and could contribute to the bilateral reduction of amygdala size observed in patients diagnosed with both TBI and PTSD (Depue et al., 2014; Stevens et al., 2016). A study in collegiate football players report a positive correlation between amygdala shape and mood states (Cho et al., 2018). TBI survivors with major depressive disorder had smaller lateral and dorsal prefrontal cortex, which mediates ventral-limbic and paralimbic pathways and influences amygdala circuitry (Jorge et al., 2004). Preclinically, diffuse and focal experimental models of TBI also identify alterations in amygdala circuitry, including increased neuronal hyperexcitability and GABA production proteins in the absence of overt neuropathology (Hallam et al., 2004; Meyer et al., 2012). In addition, pyramidal and stellate neurons in the basolateral amygdala (BLA) demonstrate increased complexity distal and proximal to the soma as early as 1 day post-injury (DPI) and persisting until at least 28 DPI indicating BLA- central nucleus of the amygdala (CeA) circuit reorganization after dTBI (Hoffman et al., 2017). Together, these data indicate that TBI-induced changes to the amygdala and associated limbic system circuitry could contribute to the development of affective disorders following injury.

Limbic system circuitry influences affective symptoms with evidence that glutamatergic neurons originating in the BLA and projecting into the CeA play a critical role in mediating anxiety-like behavior (Swanson and Petrovich, 1998; Tye et al., 2011; Janak and Tye, 2015; Babaev et al., 2018). BLA neurons are 90% glutamatergic, highlighting the role of glutamate neurotransmission in displays of affective behavior (Carlsen, 1988; Smith and Pare, 1994). Glutamate neurotransmission is regulated by astrocytes and microglia processes surrounding the synapse (Murugan et al., 2013). Glutamate transporters, Glt-1 and GLAST (located on astrocytes), rapidly remove glutamate from the extracellular space, restricting glutamate to the synaptic cleft (Danbolt, 2001; Pal, 2018). When spillover does occur, feedback through the metabotropic glutamate receptor 2 (mGluR2) can reduce presynaptic release of glutamate (Loane et al., 2012). Decreased levels of brain derived neurotropic factor (BDNF), tropomyosin-related kinase B (TrkB) receptors, and glucocorticoid receptors (GluR) – commonly reported with affective disorders – have also been reported to attenuate presynaptic glutamate release (Numakawa et al., 2009; Chiba et al., 2012; Meis et al., 2012). Each of these molecular components contribute to the complex regulation of glutamatergic neurotransmission and work to maintain homeostatic communication.

We hypothesize that TBI-induced anxiety-like behavior is mediated by changes in glutamate neurotransmission within the amygdala. In a well-established model of dTBI that causes diffuse axonal injury (DAI) in rats, we assessed the expression of anxiety-like behavior at 1-week and 1-month post-injury using open field testing (OFT). Immediately following OFT, *in vivo* amperometric recordings in the BLA and CeA evaluated glutamate clearance kinetics and available glutamate stores as an indicator of disrupted glutamate neurotransmission. Pathological and molecular analyses of nuclei-specific changes were used to identify future therapeutic targets to mediate glutamate neurotransmission associated with amygdala circuit function.

## Materials and Methods

### Animals

A total of 69 adult, male Sprague-Dawley rats (weights 279–420 grams; age 3–4 months) (Envigo, Indianapolis, IN, United States) were used in these experiments. (29 rats for amperometry, 22 for histology, and 18 rats for protein assays). Upon arrival, rats were given a 1-week acclimation period, housed in normal 12-h light/dark cycle (Red light: 18:00 to 06:00) and allowed access to food and water *ad libitum* (Teklad 2918, Envigo, Indianapolis, IN). Rats were pair housed according to injury status (i.e., injured housed with injured) throughout the duration of the study. All procedures and animal care were conducted in compliance with an approved Institutional Animal Care and Use Committee Protocol (13–460) at the University of Arizona College of Medicine-Phoenix which is consistent with the National Institutes of Health (NIH) Guidelines for the Care and Use of Laboratory Animals.

**FIGURE 1:**
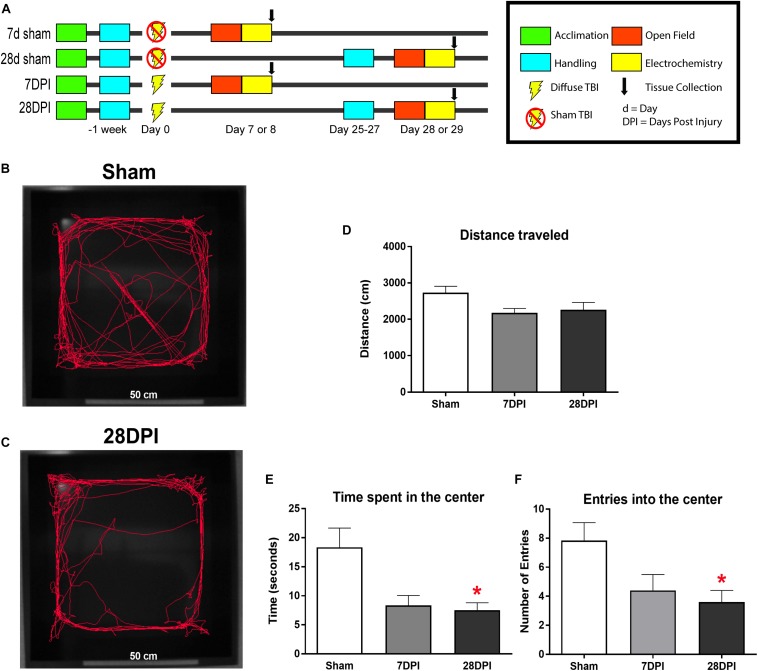
dTBI induces the expression of anxiety-like behavior. **(A)** Study design for behavioral and amperometry studies. **(B)** Representative video tracking data from Ethovision^®^ software during the first 5 min of open field testing from sham. **(C)** Representative video tracking data from Ethovision^®^ software during the first 5 min of open field testing from 28 DPI rats. **(D)** Distance traveled is similar between 7 DPI, 28 DPI, and sham [One-Way ANOVA *F*(2,29) = 3.23; *p* = 0.05]. **(E)** 28 DPI rats spent significantly less time in the center of the open field [Kruskal-Wallis *H* = 6.711; *p* < 0.05; Dunn’s *post hoc* comparison *p* < 0.05; Mann-Whitney U *post hoc p* < 0.05, *r* = 0.48]. **(F)** 28 DPI rats made significantly fewer entries into the center of the open field when compared to sham [Kruskal-Wallis *H* = 6.83; *p* < 0.05; Dunn’s *post hoc* comparison *p* < 0.05; Mann-Whitney U *post hoc p* < 0.05, *r* = 0.46]. Bar graphs represents mean ± SEM. *n* = 10–12 for each group. ^∗^*p* < 0.05 in comparison to sham.

Rats undergoing open field testing (OFT) were exposed to human contact and handled for a total of 50 min over a period of 7 days. Rats aged out to 28 DPI received an additional 30 min of handling by the same investigator over a period of 3 days immediately prior to testing to reacclimate to human contact. Handling of rats ensures that the response to the behavioral paradigm is not confounded by the threat of investigator handling (Costa et al., 2012). At 7/8 DPI or 28/29 DPI, injured and sham rats underwent OFT between 07:00 and 09:00, immediately followed by *in vivo* amperometric recordings by the handling investigator. Time of day and timing between OFT and recordings were held constant throughout all experiments to account for any potential influence of diurnal corticosterone levels and lasting molecular and structural influences from behavior (OFT)-induced stress responses (Zhang et al., 2018). For the remainder of the manuscript, DPI for the amperometric and behavioral outcome measures at 7/8 and 28/29 DPI will be abbreviated as 7 DPI and 28 DPI. For behavioral analysis and subsequent amperometric recordings, a total of 17 rats were aged to the 1-week time point (11 injured; 6 sham) and 17 rats were aged to 1-month (10 injured; 7 sham), based on *a priori* power-calculations from previous electrochemical experiments (Thomas et al., 2012) (experimental design; Figure 1A).

### Midline Fluid Percussion Injury (mFPI)

#### Surgical Procedure

Midline FPI surgery was carried out similarly to previously published methods from this laboratory by an investigator who was *not* the handler (Thomas et al., 2012). Rats were randomized into either injured or sham groups following acclimation to the vivarium facility and exposure to human contact. Briefly, rats were anesthetized with 5% isoflurane in 21% O_2_ and placed into a stereotaxic frame (Kopf Instruments, Tujunga, CA, United States) with a nose-cone that maintained 2.5% isoflurane for the duration of the procedure. A 4.8 mm circular craniotomy was centered on the sagittal suture midway between bregma and lambda carefully ensuring that the underlying dura and superior sagittal sinus were not disturbed. An injury hub created from the female portion of a 20-gauge Luer-Loc needle hub was cut, beveled and placed directly above and in-line with the craniectomy site. A stainless-steel anchoring screw was then placed into a 1 mm hand-drilled hole into the right frontal bone. The injury hub was affixed over the craniectomy using cyanoacrylate gel and methyl-methacrylate (Hygenic Corp., Akron, OH, United States) and filled with 0.9% sterile saline. The incision was then partially sutured closed on the anterior and posterior edges with 4.0 Ethilon sutures and topical lidocaine and antibiotic ointment were applied. Rats were returned to a warmed holding cage and monitored until ambulatory (approximately 60–90 min).

#### Injury Induction

Approximately 24 h following surgical procedures and the return of ambulation, rats were re-anesthetized. The hub was filled with 0.9% sterile saline and attached to the male end of a fluid percussion device (Custom Design and Fabrication, Virginia Commonwealth University, Richmond, VA, United States). After return of the pedal withdrawal reflex, an injury averaging 2.19 atm was administered by releasing the pendulum (16.5 degrees) onto the fluid-filled cylinder. Shams were attached to the fluid percussion device, but the pendulum was not released. Immediately after the administration of injury, the injury hub was removed *en bloc* and rats monitored for the presence of apnea, fencing response, and the return of righting reflex. Then, rats were briefly re-anesthetized to inspect the injury site for hematoma, herniation, and dural integrity. The injury site was then stapled closed and topical lidocaine and antibiotic ointment were applied. Inclusion criterion required that injured rats have a righting reflex time ranging from 5 to 12 min (average 7:43 ± 0:07 min) and a fencing response (McIntosh et al., 1987; Hosseini and Lifshitz, 2009). After regaining the righting reflex, rats were placed in a clean, warmed holding cage, and monitored for at least 1 h following injury before being returned to the vivarium where post-operative evaluations continued for 3 DPI. Staples for rats with a 28-day time point were removed at 7 DPI.

Midline fluid percussion injury in rats have been used for over 25 years, predominantly in the Sprague-Dawley strain. Reproducible pathophysiological and behavioral responses relevant to clinical data have been published (Riess et al., 2002; Thompson et al., 2005; Witgen et al., 2005; Lifshitz et al., 2007; Hosseini and Lifshitz, 2009; Hall and Lifshitz, 2010; McNamara et al., 2010; Alder et al., 2011; Cao et al., 2012; Learoyd and Lifshitz, 2012; Lifshitz and Lisembee, 2012; Thomas et al., 2012; Rowe et al., 2013, 2014; Harrison et al., 2014, 2015; Fenn et al., 2015). mFPI best models closed head injury with decompressive craniectomy by reproducing diffuse axonal injury without contusion or cavitation encompassing the hallmark pathology of clinical diffuse TBI. While craniectomy is a procedure often carried out in more severe clinical cases in which cerebral herniation is a concern, it provides control of increased intracranial pressure here, which when unregulated can induce a more severe injury with worse outcome (Lafrenaye et al., 2014). After regaining reflexive responses (5–12 min), injured rats require little to no medical intervention in the post-operative period, most similar to mild TBI as defined by a Glasgow Coma Score of 13–15. A more detailed discussion in the clinical relevance of mFPI can be found in our recently published review article (Lifshitz et al., 2016).

#### Open Field Testing

To evaluate for the presence of the development of affective behaviors following diffuse TBI as modeled by mFPI, an open field analysis was performed. Each rat was placed into the center of a novel black open field (measuring 69 cm × 69 cm) facing the same direction at least 1 h following the light cycle change from red to white light. White overhead lights ensured equal illumination throughout the container for a total of 15 min. Ambient white noise (60–70 decibels) in the room mitigated any variation in noise levels. Tracking was accomplished by Ethovision^®^ Software which mapped the rat directly overhead. Two regions were defined using Ethovision^®^, separating the open field into an outer region and an inner region (35 cm × 35 cm). Time spent in each zone was calculated based on the center-point of the rat. Primary outcome measures analyzed total distance traveled, time spent in the center, and entries to the center of the open field for the first 5 min of the task to be more representative of anxiety-like behavior (Gould et al., 2009).

### Electrochemistry

#### Enzyme-Based Microelectrode Arrays

Ceramic-based microelectrode arrays (MEA) encompassing four platinum (Pt) recording surfaces (15 μm × 333 μm) aligned in a dual, paired configuration were prepared to measure glutamate for *in vivo* anesthetized recordings (S2 configuration; Quanteon, Nicholasville, KY, United States). MEAs were fabricated, selected for recordings, and made glutamate sensitive as previously described (Thomas et al., 2012, 2017). Briefly, Pt sites 1 and 2 were coated with a solution containing glutamate oxidase (GluOx), bovine serum albumin (BSA), and glutaraldehyde, enabling these sites to selectively detect glutamate levels with low limits of detection (Nickell et al., 2005). Pt sites 3 and 4 were coated with only BSA and glutaraldehyde and served as sentinels, recording everything channels 1 and 2 recorded except for glutamate (Burmeister and Gerhardt, 2001). Prior to calibration and *in vivo* recordings, all four Pt recording sites were electroplated with a size exclusion layer of 1,3-phenylendediamine (mPD) (Acros Organics, Morris Plains, NJ, United States). GluOx converts glutamate into α-ketoglutarate and peroxide (H_2_O_2_). The H_2_O_2_ functions as a reporter molecule, traversing the mPD layer and is readily oxidized and recorded as current using the FAST-16 mkIII system (Fast Analytical Sensor Technology Mark III, Quanteon, LLC, Nicholasville, KY, United States) (Supplementary Figure S1).

#### Microelectrode Array Calibration

**FIGURE 2:**
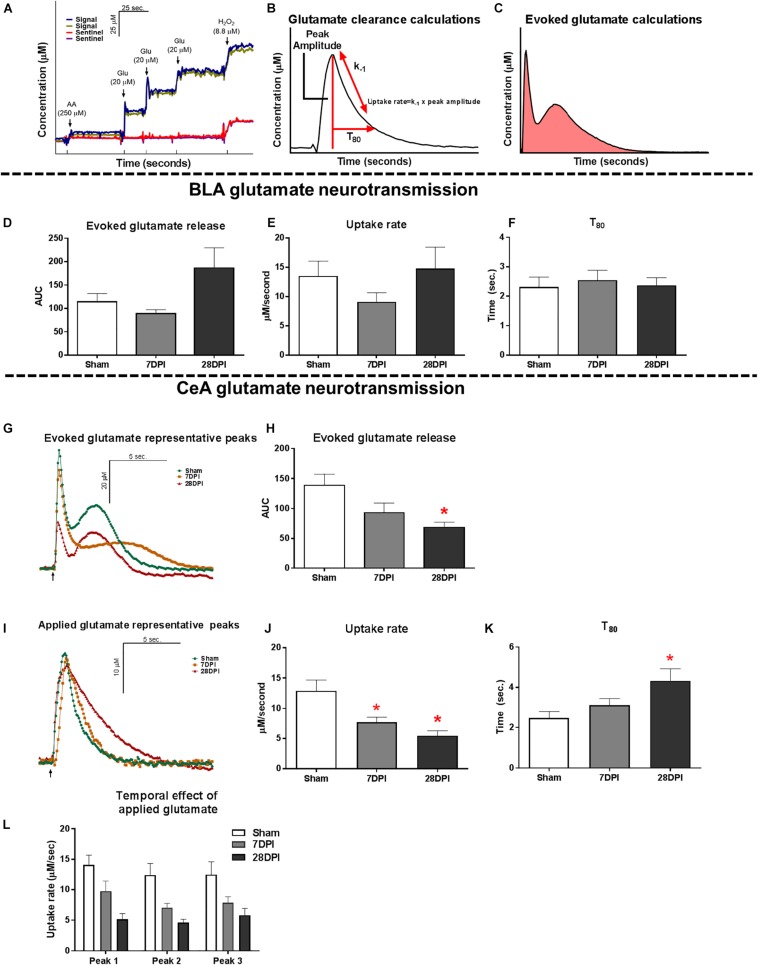
TBI induces altered glutamate neurotransmission in the CeA. **(A)** Representative calibration of glutamate selective MEA. Arrows represent aliquots of solution of either 250 μM ascorbic acid (AA), 20 μM glutamate (Glu), or 8.8 μM H_2_O_2_. **(B)** Calculations for extracellular clearance of glutamate following local applications of 100 μM glutamate. **(C)** Calculations for total evoked glutamate release following local applications of 120 mM potassium chloride solution (KCl). **(D)** No significant differences were observed in evoked glutamate release in the BLA [Kruskal Wallis *H* = 4.63; *p* = 0.10; *n* = 8–11]. **(E)** No changes in glutamate clearance were observed when assessing the uptake rate [One-way ANOVA *F*(2,21) = 1.24; *p* = 0.31] or **(F)** *T*_80_ [One-way ANOVA *F*(2,21) = 0.14; *p* = 0.87; *n* = 6–10]. **(G)** Representative traces of KCl-evoked glutamate release. Baseline levels have been adjusted to show comparison amongst the three time points. Arrow represents 120 mM KCl administration. **(H)** 28 DPI rats had 50% less total evoked glutamate when compared to shams [One way ANOVA *F*(2,19) = 4.74 *p* < 0.05 Dunnett’s *post hoc p* < 0.05, η^2^ = 0.33; *n* = 6–9] in the CeA. **(I)** Representative traces of extracellular glutamate clearance. Baselines levels have been adjusted to show comparison amongst the three time points. Arrow represents 100 μM glutamate administration. **(J)** 7 and 28 DPI rats had 40% and 58% slower uptake rate, respectively [One-way ANOVA *F*(2,22) = 7.88; *p* < 0.01, @ 7 DPI Dunnett’s *post hoc p* < 0.05, @ 28 DPI Dunnett’s *post hoc p* < 0.01, η^2^ = 0.41; *n* = 7–10]. **(K)** 28 DPI rats had a 43% increase in *T*_80_ [One-way ANOVA *F*(2,22) = 5.00; *p* < 0.05, Dunnett’s *post hoc p* < 0.01, η^2^ = 0.33; *n* = 7–10]. **(L)** Uptake rate remained consistent between subsequent additions of glutamate to the CeA [RM Two-way ANOVA *F*(2,21) = 8.16; *p* < 0.01; *n* = 7–10]. Bar graphs represents mean ± SEM. ^∗^*p* < 0.05 in comparison to sham.

On the morning of *in vivo* recordings, each MEA was calibrated *in vitro* to determine recording parameters: slope (sensitivity to glutamate), limit of detection (LOD; lowest amount of glutamate to be reliably recorded), and selectivity (ratio of glutamate to ascorbic acid). For calibration, aliquots from stock solutions were added to 40 mL of 0.05 M phosphate buffered saline (PBS) (pH 7.1–7.4; stirring; 37°C) in the following sequence: 500 μL of 20 mM ascorbic acid, three additions of 40 μL of 20 mM l-glutamate, and 40 μL of 8.8 mM H_2_O_2_ to produce a final concentration of 250 μM AA, 20, 40, and 60 μM glutamate, and 8.8 μM H_2_O_2_. A representative MEA calibration is shown in Figure 2A. For the study, a total of 48 MEAs were used with a total of 91 recording sites. Additionally, there was an overall average slope of 6.9 pA/μM, a LOD of 2.4 μM, and a selectivity of 44 to 1.

#### Microelectrode Array/Micropipette Assembly

Following calibration, a single micropipette was attached to the MEA using the following steps to allow for the local application of solutions during *in vivo* experiments. A single-barreled glass capillary with filament (1.0 mm × 0.58 mm, 6” A-M Systems, Inc., Sequim, WA, United States) was pulled using a Kopf Pipette Puller (David Kopf Instruments, Tujunga, CA, United States). Using a microscope with a calibrated reticle, the pulled micropipette was bumped against a glass rod to have an inner diameter of 7–13 μm (10.5 μm ± 0.2). Clay was used to place the tip of the micropipette between the 4 Pt recording sites. This alignment was secured using Sticky Wax (Kerr Manufacturing Co). The final measurements were the distance between the micropipette tip and the MEA surface (72 ± 3 μm) and the distance between the micropipette tip and the MEA tip (498 ± 4 μm).

#### Surgery for Amperometric Recordings

Immediately after OFT, sham and brain-injured rats were anesthetized with three or four intraperitoneal injections of 25% urethane in 15-min intervals (1.5 g/Kg; Sigma Aldrich, St. Louis, MO, United States). Following cessation of a pedal withdrawal reflex, each rat was then placed into a stereotaxic frame (David Kopf Instruments) with non-terminal ear bars. Body temperature was maintained at 37°C with isothermal heating pads (Braintree Scientific, Braintree, MA, United States). A midline incision was made and the skin, fascia, and temporal muscles were reflected to expose the skull. A bilateral craniectomy exposed the stereotaxic coordinates for the BLA and CeA. Dura was then removed prior to the implantation of the MEA. Brain tissue was kept moist through the application of saline soaked cotton balls and gauze. Finally, using blunt dissection, an Ag/AgCl coated reference electrode wire was placed in a subcutaneous pocket on the dorsal side of the subject (Moussy and Harrison, 1994; Quintero et al., 2007).

#### *In vivo* Amperometric Recording

Amperometric recordings performed here were done similar to previous published methods (Hinzman et al., 2010; Thomas et al., 2012). Briefly, a constant voltage was applied to the MEA using the FAST-16 mkIII recording system. *In vivo* recordings were performed at an applied potential of +0.7 V compared to the Ag/AgCl reference electrode. All data were recorded at a frequency of 10 Hz, amplified by the headstage (2 pA/mV) without signal processing or filtering of the data.

Immediately prior to implantation of the MEA, the pipette was then filled with 120 mM KCl (120 mM KCl, 29mM NaCl, 2.5mM CaCl_2_ in ddH_2_O, and pH 7.2 to 7.5) or 100 μM l-glutamate (100 μM l-glutamate in 0.9% sterile saline pH 7.2–7.6). Concentrations for both solutions have been previously shown to elicit reproducible KCl-evoked glutamate release or exogenous glutamate peaks (Burmeister et al., 2002; Thomas et al., 2012). Solutions were filtered through a 0.20 μm sterile syringe filter (Sarstedt AG & Co., Numbrecht, Germany) and loaded into the affixed micropipette using a 4-inch, 30-gauge stainless steel needle with a beveled tip (Popper and Son, Inc, NY, United States). The open end of the micropipette was connected to a Picospritzer III (Parker-Hannin Corp., General Valve Corporation, Mayfield Heights, OH, United States) with settings to dispense nanoliter quantities of fluid over a 1 s period using the necessary pressure of nitrogen (inert) gas using a dissecting microscope (Meiji Techno, San Jose, CA, United States) with a calibrated reticle in the eyepiece (Cass et al., 1992; Friedemann and Gerhardt, 1992).

The MEA-micropipette was localized in the BLA (A/P: ± 2.4 mm; M/L: ± 5.1 mm; D/V: −8.0 mm) and CeA (A/P: −2.4 mm; M/L: ± 3.9 mm; D/V: −8.0 mm) using coordinates from George Paxinos and Charles Watson Rat Brain Atlas (6th Edition) through use of the Dual Precise Small Animal Stereotaxic Frame (Kopf, model 962). Glutamate and KCl-evoked measures were recorded in both hemispheres in a randomized and balanced experimental design to mitigate possible hemispheric variations or effect of anesthesia duration.

#### KCl-Evoked Release of Glutamate Analysis Parameters

Once the electrochemical signal had reached baseline, 120 mM KCl was locally applied (BLA: 110 ± 8 nl; CeA: 105 ± 4 nl) to produce an evoked glutamate release. Additional ejections of KCl were completed at 2-min intervals and were volume matched at the time of administration. Criteria for analysis required that the peak with the largest amplitude was acquired from the first local application of KCl. This ensured that the data chosen were most representative of the maximum glutamate released within the surrounding neuronal tissue. Primary outcome measures were area under the curve as a proxy to investigate the total glutamate release capable of the recorded region. For a diagrammatic representation of these calculations, see Figure 2C.

#### Glutamate Clearance Analysis Parameters

Once the baseline was reached and maintained for at least 2 min (10–20 min), 100 μM glutamate was locally applied into the extracellular space (BLA: 73 ± 17 nl; CeA: 78 ± 15 nl). Exogenous glutamate was released at 30 s intervals and amplitude was matched at the time of administration. In analysis, three peaks were selected based on a predetermined amplitude range of 15 to 25 μM to ensure that data chosen were most representative of the glutamate clearance of similar volume and amplitude in accordance with Michaelis-Menton kinetics clearance parameters. The parameters for the 3 peaks were then averaged to create a single representative value per recorded region per rat. Primary outcome measures analyzed the uptake rate and the time taken for 80% of the maximum amplitude of glutamate to clear the extracellular space (*T*_80_). The uptake rate was calculated using the uptake rate constant (k_–__1_) multiplied by the peak’s maximum amplitude, thus controlling for any variation between the amount of applied glutamate between peaks. For a diagrammatic representation of these calculations, see Figure 2B.

#### MEA Placement Verification

Immediately following *in vivo* anesthetized recordings, rats were transcardially perfused with PBS followed directly by 4% paraformaldehyde (PFA). Brains were cryoprotected and sectioned at 40 μm sections to confirm MEA electrode placement. Of these, 1.96% of electrode tracts were excluded due to inaccurate placement. Representative image shown in Supplementary Figure S2.

#### Microglia Skeleton Analysis

At 1, 7, and 28 DPI, sham and brain-injured rats (*n* = 3/time point) were administered a lethal dose of Euthasol^®^ (a sodium pentobarbital mixture, 200 mg/kg, i.p., Virbac AH, Inc.) and were transcardially perfused with PBS, followed by a fixative solution containing 4% paraformaldehyde. Brains were then shipped to Neuroscience Associates Inc (Knoxville, TN) where they were embedded into a single gelatin block (MultiBrain^®^ Technology, NeuroScience Associates, Knoxville, TN, United States). Forty-micron thick sections were taken in the coronal plane and wet-mounted on 2%-gelatin-subbed slides before being stained with ionized calcium binding adaptor molecule (Iba1) primary antibody and 3,3′-Diaminobenzidine (DAB) visualization (NeuroScience Associates, Knoxville, TN, United States) to identify all microglia. Images and analysis of alternate regions of the same histology (for all NSA tissue) has been previously described (Cao et al., 2012; Lifshitz and Lisembee, 2012; Miremami et al., 2014; Rowe et al., 2016; Hoffman et al., 2017; Morrison et al., 2017; Thomas et al., 2018).

Photomicrographs of the BLA and CeA were taken using a Zeiss microscope (Imager A2; Carl Zeiss, Jena, Germany) in bright-field mode with a digital camera using a 40× objective. One digital photomicrograph in each region was acquired from the left and right hemisphere, respectively, for each time point across three coronal sections for each mFPI and sham rat, for a total of 6 images per rat. A computer-aided skeletal analysis method was used to quantify morphological remodeling of ramified microglia after experimental diffuse brain injury (Young and Morrison, 2018).

#### Skeleton Analysis

Microglia were analyzed by an investigator blinded to injury status using computer-aided skeleton analysis as previously published (Young and Morrison, 2018). Briefly, photomicrographs were converted to binary images which were skeletonized using ImageJ software (National Institutes of Health^1^) (Supplementary Figure S3). The Analyze Skeleton Plugin (developed by and maintained here^2^) was applied to the skeletonized images, which tags branches and endpoints and provides the total length of branches and total number of endpoints for each photomicrograph. Cell somas were manually counted by 2 investigators and averaged for each photomicrograph. The total branch length and number of process endpoints were normalized to number of microglia cell somas per image. Data from the 6 images were averaged to a single representative measure per animal.

#### GFAP Analysis

At 7 and 28 DPI, injured and sham rats were given a lethal dose of Euthasol^®^ and transcardially perfused with 4% PFA/PBS (sham = 3, 7 DPI = 3, 28 DPI = 4). The brains were then cryoprotected in graded sucrose solutions (15% and 30%) and cryosectioned (20 μm). Sections were prepared exactly as previously described using rabbit anti-GFAP primary antibody (1:1000; Dako, catalog# Z0334) and biotinylated horse anti-rabbit secondary antibody (1:250; Vector Laboratories, Burlingame, CA, United States; Catalog#Ba-1100) then incubated with DAB (Rowe et al., 2016; Hoffman et al., 2017). The sections were dehydrated with ethanol and cleared in citriSolv before being cover-slipped with DPX.

Montaged images encompassing both hemispheres were taken using a Zeiss microscope (Imager A2; Carl Zeiss, Jena, Germany) in bright-field mode with a digital camera. Quantitative analysis was performed using ImageJ Software (3.1.1v, NIH, Bethesda, MD, United States) on a Macintosh computer (OSX 10.11.6). The CeA was identified and traced based on topographical localization to the optic chiasm, rhinal fissure, and commissural stria terminalis. Grayscale digital images were then digitally thresholded to separate positive-stained pixels from unstained pixels. The percentage of argyrophilic (black) stained pixels was calculated for each image using the following formula:

Total area measured black/Total area measured×100=Percentage area with argyrophilic stain

Five to six rat brain hemispheres were analyzed per rat (*n* = 3–4/time point), depending on the quality of the section mounted (no folds or tearing within the area of interest). The same image was analyzed three times. The standard deviation between the percent area black was below 10% within each rat.

#### deOlmos Silver Stain Analysis

Alternating sections of brains prepared by NeuroScience Associates Inc (Knoxville, TN, United States) (see Iba1 staining methods; Thomas et al., 2018) were cryosectioned, mounted, stained using de Olmos aminocupric silver technique, counterstained with Neutral Red, and cover-slipped. Densitometric quantitative analysis was performed identical to GFAP analysis. Four hemispheres per rat (*n* = 3/time point) were analyzed and each hemisphere was analyzed three times. These 12 statistical numbers indicating the percentage of black pixels were then averaged together to a single value (per rat) and subsequently used in statistical analysis. The standard deviation between the percent area black was below 10% within each rat.

#### Tissue Dissection and Protein Extraction

At 28 DPI, a new cohort of rats (*n* = 5/each) were given a lethal dose of Euthasol^®^. Animals were transcardially perfused with ice-cold PBS for 3 min. The brain was rapidly removed and rinsed with ice-cold PBS. Tissue biopsies (1 mm diameter) taken bilaterally from the BLA and CeA were collected from 2 mm thick coronal sections made using a chilled rat brain matrix. Tissue biopsies were flash frozen and stored at −80°C until protein was extracted for automated capillary western analysis. Total protein was extracted from the BLA and CeA previously stored at −80°C. Tissues were homogenized in 250 μl of ice-cold extraction buffer (pH 8.0) containing 0.24 M Tris, 0.74 M NaCl, 100 μl TritonX100 with a protease inhibitor cocktail (complete, Roche Diagnostics; #11836153001). Tissue was homogenized with the Precellys^®^24 machine (Bertin Technologies, Montigny le Bretonneux, France) for 40 s bouts until the solution was completely clear (being chilled on iced for 2 min between bouts). Samples were then centrifuged at 3,000 × *g* for 15 min and the supernatant stored in 10–20 μl aliquots at −80C until analysis. Protein concentrations were determined using the bicinchoninic acid assay (BCA) following manufacturer’s instructions (Pierce, Rockford, IL, United States).

#### Automated Capillary Western – ProteinSimple (Wes)

Protein expression was evaluated using automated capillary western (ProteinSimple^TM^, Biotechne, San Jose, CA, United States). Experiments were run per manufacturer’s instructions and using products purchased through ProteinSimple^TM^ (unless otherwise noted), including 12–230 kDA capillary cartridges (SM-W004) and anti-rabbit detection modules (DM001). Proprietary mixtures included a biotinylated ladder and molecular weight standards as internal controls. Technical properties of the Simple Western^TM^ are described by Rustandi et al. and Loughney et al. (2014). Prior to experiments, optimization was carried out for protein concentration, primary antibody concentration, multiplexing with a biological control (GAPDH), denaturing process, and exposure time (Supplementary Table S1). Target protein concentration was chosen within the midpoint of the slope (0.125–2.5 μg/capillary). Antibody concentration was based on evidence of antigen saturation at the chosen protein concentration. Target and GAPDH (loading control) were optimized for multiplexing to ensure chemiluminescence was within systems threshold and to ensure no protein-protein interactions. If chemiluminescent detection was above the threshold, secondary antibodies from Jackson ImmunoResearch Laboratories, Inc (West Grove, PA, United States) were used to dilute the manufacturer’s secondary antibody to reduce amplification of highly expressed targets (Goat Anti-Rabbit 111-005-045, HRP-Goat Anti-Rabbit 111-035-045, Goat Anti-Mouse 115-005-062, and HRP-Goat Anti-Mouse 115-035-062). Denaturing temperature was chosen based on low signal to noise and baseline. The high-dynamic range of the exposures (algorithm in software) was used for data analysis in all experiments. Every capillary cartridge (25 capillaries) was run with the following controls: the same brain homogenate as a positive control, Erk as a system control, Antibody only, and protein only.

Protein extracts from amygdala samples were combined with sample buffer and master-mix (40 mM DTT, 0.1× ProteinSimple Sample Buffer, and 1× Fluorescent Standards) to achieve the desired protein concentration. Samples were then denatured via heating block at the optimized temperature (37°C × 30 min). Duplicate capillaries of each sample were loaded per manufacturer’s instructions, the cartridge was centrifuged at 2500 RPM for 5 min, and placed into the automated capillary western machine where proteins were separated by size (electrophoresis), immobilized, and immunoprobed in individual capillaries. Once loaded into the instrument, the standard default metrics recommended by ProteinSimple were utilized for separation, incubation, and detection. The associated software, Compass (ProteinSimple^®^), generates an electropherogram with peaks corresponding to the expression of proteins of interest and calculates the area under the curve (AUC) for each peak (Figure 4E). To calculate relative protein expression, the AUC for the protein was normalized to the AUC for the biological control (GAPDH). The ratios from duplicate capillaries were averaged. Then, ratios from injured animals were normalized to shams in the same capillary cartridge.

#### Statistical Analysis

Based on KCl-evoked glutamate release in the thalamus from a previous publication by Thomas et al (Thomas et al., 2012), 3–12 rats per group could detect a 100% increase in evoked glutamate release at 80% power at a significance level of 0.05 on a 2-sided test. Statistical analysis was determined by *a priori* planned comparisons, where our primary outcome measure (OFT, electrochemistry, histology, protein levels) was to detect changes between 7 DPI or 28 DPI and shams. All data were analyzed using a customized Microsoft Excel^®^ spreadsheet and GraphPad^®^ software. Seven day and 28 day shams did not statistically differ from one another for all outcomes of behavior (e.g., time in center: 7D sham 31.3 ± 7.2 s, 28D sham 34.7 ± 6.7 s) and electrochemical measures (e.g., evoked glutamate release (AUC): BLA-7D sham 105.9 ± 33.9, 28D sham 119.1 ± 20.8; and CeA-7D sham 136.4 ± 34.2 and 140 ± 23.6). Therefore, 7D and 28D shams were combined for statistical analysis. When ANOVA assumptions were met and the dependent variable was continuous, data were analyzed using a one-way ANOVA with Dunnet’s *post hoc* comparison to sham. All data were assessed for normality and variability. Data not passing Shapiro-Wilks normality tests or Brown-Forsythe variability test were analyzed as non-parametric statistics with a Kruskal-Wallis (KW) analysis and Dunn’s *post hoc* comparison to sham. Outliers in the open field test were determined using three standard deviations from the mean. Using these metrics, two rats were excluded from consideration (7-day sham, 7 DPI). Amperometric measures from the paired MEA recording sites for each outcome in each region were averaged and used as a single data point. Outliers for amperometric analysis were determined using a ROUT test with Q = 5% (BLA = 1 of 28; CeA = 4 of 28). Additional analysis of extracellular clearance of glutamate in the CeA assessing for the potential effects of multiple additions of glutamate and differential effects of nuclei-specific microglial activation were conducted with a two-way ANOVA with a Tukey comparison. To be included in the amperometric recordings, the rat had to be included in the in open field testing. Molecular data for glutamate transporters were analyzed using an unpaired, two-tailed Student’s *t*-test. Based on our hypothesis of a decrease in protein levels for BDNF, TrkB, and GluR, we used a one-tailed Student’s *t*-test. For all instances, statistical significance was defined at *p* < 0.05.

#### Effect Size

For parametric calculation using the one-way ANOVA, eta squared η^2^ was conducted as described by Lakens (2013). η^2^ was evaluated and reported such that 0.02 represents a small effect size, 0.13 represents a medium effect size and 0.16 represents a large effect size. Data comparing two means and analyzed using a Student’s *t*-test were evaluated using Cohen’s d. For non-parametric calculations using the Kruskal-Wallis test, Pearson’s r was reported as described by Fritz et al. (2012). To do so, a Mann Whitney U *post hoc* comparison was done between the two groups identified to have a difference as indicated by the Dunn’s *post hoc* comparison. Then a Pearson’s r effect size calculation was evaluated and reported such that a 0.1 equals a small effect size, 0.3 represents a medium effect size and 0.5 represents a large effect size.

#### Data Analysis Validation

All outcome measures were evaluated for variability based on cohort membership, cage-mates, cage changes, time/order of open field testing, and time of recordings. Of these, no variables, other than injury status, could explain the variation observed in the presented data sets.

## Results

### dTBI Induces the Expression of Anxiety-Like Behavior

Representative traces of first 5 min of open field exploration illustrate behavioral performance between brain-injured and sham rats (Figures 1B,C). Distances traveled at 7 DPI and 28 DPI did not reach significance when compared to shams [*F*(2,29) = 3.23; *p* = 0.054; Figure 1D]. Rats at 7 DPI did not significantly alter their duration in the center of the open field, whereas rats at 28 DPI spent 57% less time in the center when compared to shams (KW = 6.71; *p* < 0.05; Dunn’s *post hoc p* < 0.05; *r* = 0.48; Figure 1E). Rats at 28 DPI also made 54% fewer entries into the center of the open field when compared to shams (KW = 6.83, *p* < 0.05; Dunn’s *post hoc p* < 0.05; *r* = 0.46; Figure 1F). These data indicate that mFPI results in the expression of anxiety-like behavior by 28 DPI.

### Glutamate Neurotransmission in the BLA Does Not Change Over 1-Month Following dTBI

As the main sensory nuclei for anxiety-like behavior, the BLA was examined for alterations of glutamate neurotransmission (Pitkanen et al., 1997; Phelps and LeDoux, 2005). BLA tissue was depolarized using a local application of isotonic 120 mM KCl (75–150 nL) to evoke the release of neurotransmitters. Similar volumes of KCl were applied to the extracellular space (Supplementary Figure S4A). KCl-evoked glutamate release in the BLA was not significantly different between 7 DPI and 28 DPI brain-injured rats when compared to shams (KW = 4.63 *p* = 0.10; Figure 2D).

Evaluation of BLA extracellular glutamate clearance parameters was accomplished through local application of 100 μM exogenous glutamate. Peaks were amplitude-matched to control for Michaelis-Menten kinetics, then analyzed for glutamate clearance parameters (Supplementary Figure S4B). Uptake rate was unaltered within the BLA at 7 DPI or 28 DPI following injury when compared to shams [*F*(2,21) = 1.24; *p* = 0.31; Figure 2E]. Furthermore, time taken for 80% of maximum applied glutamate to clear (*T*_80_) was not altered within the BLA at 7 DPI or 28 DPI when compared to shams [*F*(2,21) = 0.140; *p* = 0.870; Figure 2F]. These data indicate that evoked glutamate release and glutamate clearance parameters were not influenced by mFPI at 7 DPI or 28 DPI.

### Glutamate Neurotransmission in the CeA Is Significantly Altered Over 1-Month Following dTBI

The CeA is the main amygdaloid nucleus for regulating anxiety-like behavior (Pitkanen et al., 1997; Phelps and LeDoux, 2005; Tye et al., 2011). Surrounding tissues, including pre-synaptic terminals along the BLA-CeA axis, were depolarized to evoke the release of glutamate using local application of 75–150 nL of 120 mM isotonic KCl. There were no statistically significant differences in the volume of applied KCl (Supplementary Figure S4D). Representative traces of KCl-evoked glutamate release are shown in Figure 2G. Glutamate released in CeA was 50% less at 28 DPI when compared to shams [*F*(2,19) = 4.74; *p* < 0.05; Dunnett’s *post hoc p* < 0.05; η^2^ = 0.33; Figure 2H]. Initial peak amplitude was also significantly less at 28 DPI when compared to shams [*F*(2,19) = 4.89; *p* < 0.05; Dunnett’s *post hoc p* < 0.05; η^2^ = 0.34; Supplementary Figures S4C,F].

Evaluation of CeA extracellular glutamate clearance was accomplished through local application of 100 μM exogenous glutamate. Representative traces of resulting glutamate peaks chosen for analysis are shown in Figure 2I. Peaks were amplitude-matched to control for Michaelis-Menten kinetics, then analyzed for glutamate clearance parameters (Supplementary Figure S4E). Uptake rate was significantly decreased at 7 DPI and 28 DPI when compared to shams resulting in 40% and 58% decreases, respectively [*F*(2,22) = 7.88; *p* < 0.01; Dunnett’s *post hoc p* < 0.05 for 7 DPI and *p* < 0.01 for 28 DPI; η^2^ = 0.42; Figure 2J]. Further, *T*_80_ of 28 DPI rats was 43% longer when compared to shams [*F*(2,22) = 5.00; *p* < 0.05; Dunnett’s *post hoc p* < 0.01; η^2^ = 0.31; Figure 2K]. Uptake was independent of subsequent local applications of glutamate to the CeA [RM *F*(2,21) = 8.16; *p* < 0.01; Figure 2L]. These data indicate that evoked glutamate release and glutamate clearance parameters in the CeA were significantly influenced by mFPI over 28 DPI.

### Nuclei-Specific Microglia Deramification Manifests Acutely Along BLA-CeA Axis Following dTBI

**FIGURE 3:**
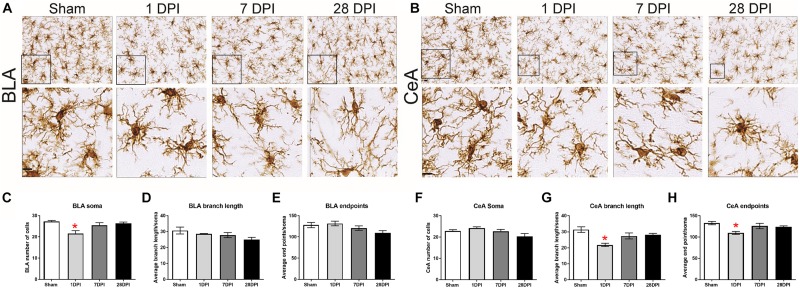
Acute, nuclei-specific microglial alterations occur following dTBI. Representative images of Iba1 staining in the **(A)** BLA and **(B)** CeA. Cropped photomicrographs correspond to the highlighted square (Scale bars = 10 μm). **(C)** In the BLA, the number of microglia changed over time post-injury [One-way ANOVA *F*(3,8) = 6.32; *p* < 0.05; η^2^ = 0.70]. **(D)** Branch length (μm/cell) or **(E)** number of microglia process endpoints per cell did not significantly change over time [One-way ANOVA *F*(3,8) = 2.32; *p* = 0.15 and One-way ANOVA *F*(3,8) = 3.18; *p* = 0.09]. **(F)** In the CeA, the average number of microglia per field did not significantly change over time [One-way ANOVA *F*(3,8) = 3.17; *p* = 0.09]. **(G)** Contrasting to the BLA, branch length (μm/cell) [One-way ANOVA *F*(3,8) = 5.27; p < 0.05; η^2^ = 0.66]. **(H)** Number of microglia process endpoints per cell were significantly decreased at 1 DPI in comparison to sham [One-way ANOVA *F*(3,8) = 7.32; *p* < 0.05; η^2^ = 0.73]. Bar graphs represents mean ± SEM. *n* = 3 for each group. ^∗^*p* < 0.05 in comparison to sham.

Images of microglia are depicted for BLA (Figure 3A) and CeA (Figure 3B). In the BLA, microglial cell count significantly changed as a function of time, decreasing at 1 DPI [*F*(3,8) = 6.32; *p* < 0.05; Dunnett’s *post hoc p* < 0.01 Figure 3C]. The branch length per cell (μm/cell) did not significantly change over time [*F*(3,8) = 2.32; *p* = 0.15; η^2^ = 0.70; Figure 3D]. Furthermore, the number of microglia process endpoints per cell did not significantly change over time [*F*(3,8) = 3.18; *p* = 0.09; Figure 3E].

Identical analysis was performed on microglia in the CeA. The microglial cell count per field did not significantly change over time [*F*(3,8) = 3.17; *p* = 0.09; Figure 3F]. The branch length per cell (μm/cell) significantly decreased at 1 DPI [*F*(3,8) = 7.32; *p* < 0.05; η^2^ = 0.73; Figure 3G]. The number of microglia process endpoints per cell was also significantly decreased at 1 DPI [*F*(3,8) = 5.27; *p* < 0.05; Dunnett’s *post hoc p* < 0.05; η^2^ = 0.66; Figure 3H].

The same three variables were evaluated with a two-way ANOVA to determine whether the influence of time-post injury was similar in between the BLA and CeA. The number of cells [*F*(3,16) = 7.85; *p* < 0.05], endpoints [*F*(3,16) = 5.152; *p* < 0.05], and process length [*F*(3,16) = 3.94; *p* < 0.05] all differed between regions, indicating that TBI-induced changes in microglial morphology at 1 and 28 DPI are region dependent (Supplementary Figure S5). Although there is an acute microglial response in the CeA, similar to other regions in this injury model (Miremami et al., 2014; Morrison et al., 2017; Thomas et al., 2018), and dynamics over time are region-dependent; these data do not provide evidence that microglia have a role in mediating glutamate neurotransmission in BLA-CeA communication at 28 DPI.

### Absence of Activated Astrocytes Over 1 Month After dTBI

**FIGURE 4:**
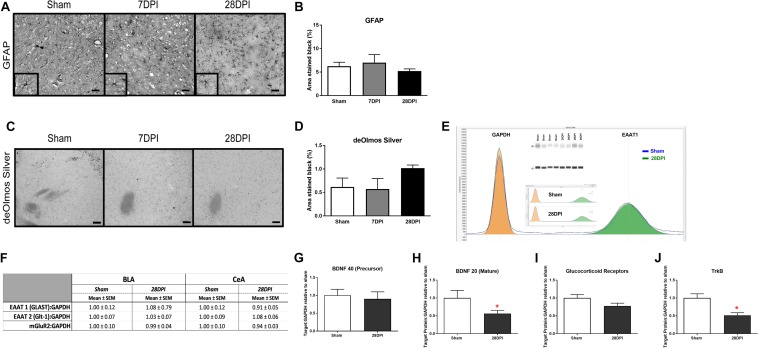
Changes in neurotransmission occur in the absence of neuropathology, astrocytosis and molecular alterations in glutamate transporters and receptor. **(A)** Representative images of GFAP staining of the CeA (40× magnification; scale bar = 50 μm). **(B)** Pixel density analysis of GFAP staining in the CeA revealed no significant differences following diffuse TBI [One-way ANOVA *F*(2,7) = 0.80; *p* = 0.49; *n* = 3*–*4 per group]. **(C)** de Olmos silver stained sections of the CeA (40× magnification; scale bar = 50 μm). **(D)** Pixel density analysis of aminocupric silver stained tissue revealed that there was no difference following diffuse TBI [One-way ANOVA *F*(2,6) = 0.22; *p* = 0.40; *n* = 3*–*4 per group]. Automated capillary western blots (in duplicate) were performed to quantify the amount of synaptic glutamate transporters (Glt-1, GLAST) and pre-synaptic glutamate receptors (mGluR2, GluR) at 28 DPI. **(E)** Representative quantification of sham and injured GLAST (EAAT1) shown individually, overlapped and with recreated western blot. **(F)** Levels of GLAST, Glt-1, and mGluR2 were similar to sham indicating an alternative mechanism for observed changes in glutamate neurotransmission (*n* = 4*–*5 per group). **(G)** The precursor protein to BDNF was similar to sham at 28 DPI. **(H)** Protein levels of mature BDNF were decreased by 43% (*t*_4_,_5_ = 2.07; *p* < 0.05; *d* = 1.33). **(I)** GluR approached significance with a 20% decrease (*t*_4_,_5_ = 1.854; *p* = 0.053). **(J)** TrkB receptors were decreased by 49% (*t*_4_,_5_ = 3.68; *p* < 0.01; *d* = 2.41). *n* = 4*–*5 per group. Bar graphs represent mean ± SEM.

Robust astrocytosis in the BLA has been previously reported at 7 DPI and resolved by 28 DPI following experimental dTBI using mFPI (Hoffman et al., 2017). Astrocytes are a major contributor to glutamate neurotransmission, where activation indicates a role in glutamate clearance from the extracellular space. GFAP intensity was analyzed as an indicator of astrocytosis in the CeA. There was no evidence that mFPI influenced astrocytosis over time in the CeA [*F*(2,7) = 0.80; *p* = 0.49; Figures 4A,B].

### Absence of Overt Neuropathology in the CeA Following dTBI

The BLA has been described as lacking overt neuropathology at 7 DPI and 28 DPI (Hoffman et al., 2017), however, the CeA has not been evaluated in a model of mFPI. Using de Olmos silver stain technique, neuropathology in the CeA was assessed at 7 DPI and 28 DPI compared to shams. No overt pathology was identified in the CeA following mFPI [*F*(2,6) = 1.99; *p* = 0.22; Figures 4C,D].

### Alteration in Glutamate Neurotransmission Are Independent of Glutamate Transporters and Pre-synaptic Receptors

Observed alterations to glutamate neurotransmission could result from altered levels of glutamate transporters or presynaptic metabotropic receptors (Ueda et al., 2001). Proteins involved in glutamate transport [EAAT1 (GLAST) EAAT2 (Glt-1)] were evaluated at 28 DPI for the BLA and CeA. Levels of GLAST and Glt-1 were similar to sham [GLAST (BLA t_8_ = 0.59 *p* = 0.57; CeA *n* = 4, t_7_ = 0.75; *p* = 0.49; Glt-1 (BLA t_7_ = 0.25; *p* = 0.81; CeA t_6_ = 0.81; *p* = 0.45)] (Figures 4E,F). Pre-synaptic metabotropic glutamatergic receptors (mGluR2) levels did not change in comparison to shams (BLA *t*_9_ = 0.09; *p* = 0.93; CeA *t*_6_ = 0.51; *p* = 0.63) (Figure 4F), indicating that observed changes in glutamate neurotransmission were independent of changes in total protein levels of glutamate transporters and mGluR2. Supplementary Figure S6 depicts a schematic showing localization of these molecules on a presynaptic terminal.

### BDNF and TrkB Decreased at 1-Month Post-dTBI

As BDNF, GluR, and TrkB, have been shown to influence presynaptic glutamate release, we tested the hypothesis that dTBI caused decreased levels of BDNF, GluR, and TrkB in the amygdala by 28 DPI (Kunugi et al., 2010). Protein levels of mature BDNF were decreased by 43% (t_7_ = 2.07; *p* < 0.05; *d* = 1.33; Figure 4H) (BDNF precursor was not different; Figure 4G) and GluR approached significance with a 20% decrease (t_7_ = 1.854; *p* = 0.053; Figure 4I). TrkB receptors were decreased by 49% (t_7_ = 3.68; *p* > 0.01; *d* = 2.41; Figure 4J). These data indicate that a single dTBI can significantly decrease BDNF and TrkB in the amygdala by 1-month post-injury, coinciding with the changes in evoked glutamate neurotransmission and expression of anxiety-like behavior.

## Discussion

Multifactorial mental illnesses are the result of poorly understood pathophysiology that confounds treatment approaches leading to poor symptom control. These are the first experiments that demonstrate dTBI initiates a cascade of molecular events in the amygdala capable of contributing to the expression of anxiety-like behavior. By 1-month post-injury, decreased glutamate release and slower glutamate clearance within the CeA coincided with the expression of anxiety-like behavior. There were no changes in the BLA. Changes in glutamate neurotransmission occurred despite similar protein levels of glutamate transporters, mGluR2, and in the absence of overt neuropathology or glial pathology. BDNF and TrkB protein levels were significantly decreased at 28 DPI, and may be a potential target to modulate glutamate signaling related to anxiety-like behavior. These data indicate novel targets in the quest for understanding chronic TBI pathophysiology associated with TBI-induced affective disorders.

Open field testing is an ethological behavioral-based test for systematic assessment of unconditioned general exploratory drive that can serve as an initial screening of emotional or anxiety-like behavior, locomotor activity, and novel environment exploration. The test can distinguish between the primal drive to survive (aversion to light and innate predatory response) versus exploration (enter the center area) by quantifying thigmotactic variables, distance traveled, entrances and time in the center of the open field as an indicator of emotionality or anxiety-like behavior (Roth and Katz, 1979; Ennaceur, 2014). Specifically, increased time spent alongside the walls of the open field enclosure is indicative of a heighten aversion response and has a high correlation factor with measures of anxiety in the elevated plus maze in rodents (Carola et al., 2002). Characterized by validated metrics reviewed by Gould et al. (2009), we show evidence of late-onset anxiety-like behavior following dTBI. By 28 DPI, rats spent less time and made fewer entries into the center of the open field when compared to shams, indicative of anxiety-like behavior. Distance traveled between sham and brain-injured rats did not reach significance as previously demonstrated in Liu et al. in the same model at the same time point (Liu et al., 2017). While Liu et al. (2017) reports dopamine axonal damage, 25% dopamine neuronal loss and microglia activation the nigrostriatal pathway, a minimum of 50% loss of substantia nigral dopamine neurons is associated with movement symptoms (Grosch et al., 2016), supporting that the loss of DA neurons is not the primary cause of decreased locomotor activity. Morris Water Maze testing at 15 DPI further provides evidence that injured rats were capable of swimming a similar distance at similar swim speed as shams indicating a behavioral response rather than an motor deficit (Clausen et al., 2017). Repeated publications from our lab support that non-noxious sensory stimulation of the whiskers significantly increases freezing, guarding, evasive behaviors and corticosterone levels at 28 DPI in comparison to sham and 7 DPI (McNamara et al., 2010; Thomas et al., 2012), supporting that the observed trends in decreased distance may be associated with a heighten aversion response.

The presence of anxiety and fear-like behavior has been reported at chronic time points in other models of TBI using OFT, elevated plus maze and fear conditioning (Kovesdi et al., 2011; Elder et al., 2012; Shultz et al., 2013; Almeida-Suhett et al., 2014; Hou et al., 2017), however, the presence of affective deficits is inconsistent and varies between experimental models, species, behavioral paradigms and labs. For these experiments, we only used a single behavioral test because repeated stressors (including behavioral tests) can also result in BLA neuronal hypertrophy and enhanced anxiety-like behavior (Vyas et al., 2004), potentially confounding electrochemical experiments. A more thorough independent assessment of anxiety, fear, and depressive behaviors at 1-month post-injury are needed to determine the extent of TBI-induced affective morbidity (Ennaceur, 2014).

**FIGURE 5:**
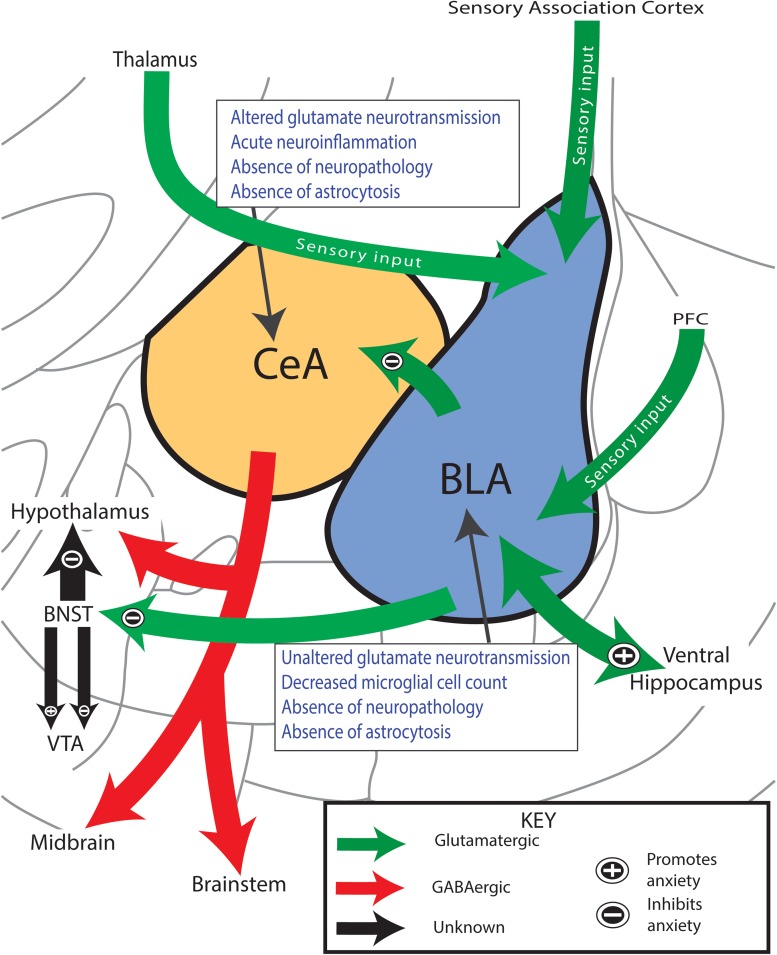
Anxiety-like behavior coincides with altered glutamate neurotransmission in amygdala circuitry. Simplified circuitry of the rat’s efferent and afferent projections of the amygdala that are considered to play important roles in the regulation of anxiety-like behavior. Glutamatergic projections enter the BLA from the PFC and thalamus and carry sensory information of the overall anxious state of the rat. The BLA interprets this information and sends efferent outputs to either promote (+) or inhibit (–) the display of anxiety-like behavior. Activation of BLA-CeA circuitry has shown to mediate anxiolytic behavior. Boxes summarize findings. PFC, prefrontal cortex; BLA, basolateral amygdala; CeA, central nucleus of the amygdala; BNST, bed nucleus stria terminalis; VTA, ventral tegmental area. Anatomy modified from Paxinos and Watson, 2007.

Glutamatergic connections between the BLA and CeA have been shown to regulate affective symptomatology during neurological assessment tasks such as open field (Tye et al., 2011; Kim et al., 2013; Janak and Tye, 2015), integrating limbic system circuitry input from numerous regions of the brain (Figure 5; Tye et al., 2011; Kim et al., 2013; Janak and Tye, 2015; Tovote et al., 2015). The BLA integrates sensory afferents from the prefrontal cortex (PFC), thalamus, and sensory association cortex and relays this information to the ventral hippocampus, bed nucleus of the stria terminalis (BNST), or CeA by projects that can either promote or diminish the expression of anxiety-like behavior (Phelps and LeDoux, 2005; Janak and Tye, 2015; Tovote et al., 2015; Hrybouski et al., 2016). Reciprocal and interconnections between and within nuclei of the amygdala not shown in our diagram also modulate, in part, the BLA-CeA circuitry and most likely influence the expression of affective behaviors (Pitkanen et al., 1997; Phelps and LeDoux, 2005). While the BLA-CeA circuit plays a known role in mediating affective behaviors (Pitkanen et al., 1997; Phelps and LeDoux, 2005); the functional, structural, and molecular components underlying the development after TBI have never been evaluated.

For real-time recordings of extracellular neurotransmission, *in vivo* amperometry coupled with glutamate selective multielectrode arrays were utilized for their excellent spatial and temporal resolution and the presence of sentinel channels to verify glutamate specificity. Localized KCl administration induced non-specific neurotransmitter release in which glutamate-sensitive MEA’s measured synaptic glutamate overflow. In the BLA, no significant changes were detected, however, in the CeA glutamate release was decreased by 28 DPI compared to uninjured shams. Reduced glutamate release indicates less stores available for potential neuronal communication between those located in presynaptic neurons, interneurons, and astrocytic processes. The second peak within the biphasic profile of KCl-evoked glutamate release (Figure 2G) provides evidence that glial transmission could be contributing to glutamate overflow whereas the first peak is thought to primarily be the response of presynaptic neurons (Parpura et al., 1994; Larter and Craig, 2005). While the amplitude of the first peaks was also significantly decreased at 28 DPI compared to shams (Supplementary Figures S4C,F), area under the curve was used to evaluate glutamate overflow. Changes in glutamate overflow could arise from impaired vesicular loading (Watt et al., 2000) or secondary to inhibitory GABA-ergic modulation (Jacob et al., 2008). We did not find an injury-induced change in the protein level of mGluR2 in the CeA, indicating that total mGluR2 is not predictive of changes in presynaptic glutamate release. We did not observe astrocytosis or perturbation of microglia at 28 DPI. Decreased BDNF/TrkB/GluR protein levels can reduce presynaptic glutamate release, however, localization, cell type, and functional studies are necessary to identify their role (Numakawa et al., 2009; Meis et al., 2012). As the majority of the CeA is GABAergic in nature, the release of glutamate is hypothesized to be from pre-synaptic neurons originating in the BLA. As Tye et al. (2011) indicates, stimulation of these synapses produces a decrease in the expression of anxiety behavior (McDonald, 1982; Carlsen, 1988; Smith and Pare, 1994). Therefore, decreased glutamate release in the CeA may be indicative of a permissive effect along BLA-CeA circuit such that the ability to suppress an adverse, anxiety-like response is diminished, as indicated by the concurrent expression of anxiety-like behavior. Thus, while novel in TBI, our results parallel the consensus that dysregulation of the CeA may be concomitant with the expression of affective conditions (Kalin et al., 2004; Etkin et al., 2009).

Locally applied glutamate to the BLA and CeA allowed us to calculate glutamate clearance from the extracellular space, primarily mediated by glutamate transporters (Glt-1 and GLAST) located on adjacent astrocytes in the rodent (Danbolt, 2001). The affinity of glutamate transporters can be evaluated using glutamate uptake rate, whereas the number of membrane bound transporters is estimated using *T*_80_ (Thomas et al., 2017). In the BLA, glutamate clearance parameters did not change as a function of injury over time. In the CeA, the uptake rate was significantly slower at 7 DPI and 28 DPI while glutamate took significantly longer to clear (*T*_80_) by 28 DPI. For calculations of glutamate parameters, three consecutive peaks in each region were analyzed. The consecutive peaks were reproducible, and the uptake rate remained significantly different regardless of the repeated applications, confirming that observed alterations to glutamate clearance are not caused by immediate tissue compensation. Changes in glutamate clearance were also not due to changes in the protein levels of glutamate transporters. Slower glutamate clearance could also be caused by surface expression, post-translational modifications, or adaption of glia and adjacent cells (Maragakis and Rothstein, 2004; Hinzman et al., 2012).

No significant alterations were detected in the BLA in anesthetized rats in this study, although studies using focal TBI models indicate that BLA circuitry becomes weakened through altered neuronal excitability, changes in N-methyl-D-aspartate (NMDA) receptors, and changes to GABAergic production proteins (GAD-67), all of which may provide compensatory responses that primarily influence glutamate release in the CeA (Reger et al., 2012; Palmer et al., 2016; Hoffman et al., 2017). Previously, we reported increased neuropathology in the somatosensory cortex and thalamus following dTBI, which could also alter sensory input into the BLA (Lisembee and Lifshitz, 2008; Thomas et al., 2018). Recent publications have also indicated subacute metabolic, and chronic metabolic and structural changes mapped directly to the CeA, and impairments in extinction of contextual fear differences at a chronic time point after experimental TBI (Zhao et al., 2018; Jaiswal et al., 2019; Kulkarni et al., 2019). In accordance with the data in this manuscript, these reports specifically identify the CeA as a vulnerable anatomic locus for future investigations, where FDA approved drugs with affinities for identified targets can be evaluated for new indications on their influence on glutamate signaling in anxiety-like behavior.

No significant differences were measured between 7 DPI and 28 DPI for both OFT and glutamate neurotransmission, however, a trend toward significance was observed over time post-injury. We’ve previously reported increased dendritic branching of both pyramidal and stellate glutamatergic neurons in the BLA at 1 DPI, continually changing and persisting out to 28 DPI, with evidence of increased distal branching at 28 DPI in comparison to sham (Hoffman et al., 2017). This continuum of increased neuromorphic connection is indicative of increased communication within and beyond the BLA, and could differentially influence behavior and glutamatergic recordings, contributing to less robust responses at 7 DPI. We have previously published a different time course of morphological changes in the ventral posteromedial nucleus of the thalamus (VPM), the thalamic relay of the somatosensory whisker barrel circuit (Thomas et al., 2018). The time course of events in the BLA are different in comparison to the VPM, where dendritic loss peaks and 7 DPI and significantly increases by 28 DPI, coinciding with increased neuropathology (silver stain), glial activation, evoked-glutamate release, and the manifestation of hypersensitivity to whisker stimulation (McNamara et al., 2010; Thomas et al., 2012, 2018). The same archival histology for neuropathology and microglia has also been published for the somatosensory barrel fields of the whisker circuit (Lisembee and Lifshitz, 2008; Cao et al., 2012; Morrison et al., 2017) and replicated in mouse mFPI (Rowe et al., 2019). Together, these data indicate that TBI-induced circuit pathophysiology is dependent on time, region, and likely, circuit connectivity.

Microglia and astrocytes can directly and indirectly contribute to the homeostasis of excitatory neural circuitry through synaptic remodeling, release of soluble molecules, microglia-astrocyte interaction, and scaling excitatory-inhibitory signaling (reviewed in Henstridge et al., 2019). Specifically, activated microglia can promote neuropathology and synaptic loss that can directly influence glutamate neurotransmission. Morphological analysis of microglia in the BLA and CeA nuclei were evaluated to identify a potential role for microglia in TBI-induced changes in glutamate neurotransmission. At 28 DPI, microglia morphology indicated a ramified state that may not contribute to the changes measured in glutamate neurotransmission. However, nuclei-dependent neuroinflammatory response did preceded changes in glutamatergic neurotransmission along the BLA-CeA circuit. De-ramified microglia were identified in the CeA, but not the BLA, at 1 DPI. In the CeA, microglial cell counts did not change over time, while the summed process length and number of microglial process endpoints per cell were found to be significantly decreased at 1 DPI. These data suggest that microglia become de-ramified in the CeA early and recover to sham levels by 7 DPI, indicative of an acute inflammatory response after injury (Ramlackhansingh et al., 2011). Targeted studies mediating acutely activated microglia are necessary to determine whether early activation influences long-term glutamate neurotransmission.

Subsequently, we analyzed CeA neuropathology to determine if axonal injury and neurodegeneration was coincident with changes in glutamate neurotransmission. However, in accordance with previous results published for the BLA (Hoffman et al., 2017), silver staining had no evidence of neuronal or axonal injury.

Glutamate neurotransmission is primarily mediated by glutamate transporters located on astrocytes. Changes in overflow of glutamate from the tripartite synapse can be attributed to altered astrocytic function. While astrocytes have been implicated in anxiety symptoms, this role is unclear. Studies report that GFAP levels can mediate expression of glutamate transporters, such that low levels of GFAP are associated with mood disorders, activation of astrocytes influence neurotransmission, and microglial activation triggers astrocyte-mediated modulation of excitatory neurotransmission (Pascual et al., 2012). In evaluating GFAP immunohistochemistry, increased levels of GFAP can be interpreted as a presence of activated astrocytes or increased number of astrocytes, whereas decreased levels can indicate a downregulation of GFAP and/or reduced number of astrocyte (reviewed in Hughes et al., 2004; Rajkowska and Miguel-Hidalgo, 2007; Zhou et al., 2019). Analysis of GFAP density in the CeA revealed no differences over time post-injury, in contrast to our previous work in the BLA, where morphology and increased GFAP staining indicated activated astrocytes at 7 DPI (Hoffman et al., 2017). Furthermore, glutamate transporter protein levels were not decreased at 28 DPI, negating a GFAP expression level/glutamate transporter interaction. These data do not support a substantial role for astrocytes in the changes in anxiety-like behavior and glutamate neurotransmission reported in this manuscript. Taking into consideration the microglia results, it is also unlikely that microglia-astrocyte interactions mediate these changes.

Chronologically, dTBI initiates an immediate release of glutamate, a stress response that includes increased circulating corticosterone levels, and a cascade of inflammatory cytokines that could mediate early CeA microglial activation through ionotropic and metabotropic glutamate receptors and glucocorticoid receptors (Murugan et al., 2013; Madalena and Lerch, 2017). We have previously reported a significant decrease in basal plasma corticosterone levels and a blunted stress response at 56 DPI, indicating chronic dysregulation of the HPA axis in this dTBI model (Rowe et al., 2016). Chronic dysregulation of corticosterone in response to stress has also been supported in other TBI models at both earlier and later time points (Griesbach et al., 2011, 2012). The central amygdala mediates the HPA axis response to stress, where changes in circulating corticosterone levels, GR, BDNF, and Trk-B receptor expression have been implicated in the pathology of affective disorders (anxiety-like behavior and posttraumatic stress disorder-like phenotypes) in addition to presynaptic glutamate signaling (Numakawa et al., 2009; Kunugi et al., 2010; Tejeda and Diaz-Guerra, 2017). Further evaluation is necessary to determine whether a role exists for corticosterone dysregulation and corticosterone regulated receptors in the development of anxiety-like behavior and changes in glutamate neurotransmission over time following dTBI.

Clinically, it is difficult to differentiate the initiating insult for patients experiencing affective symptoms, such as anxiety or PTSD, as similar pathophysiology exists between dTBI and chronic stress (Owens et al., 2008; French, 2010; MacGregor et al., 2011; DePalma and Hoffman, 2016). This study provides a novel link between dTBI-induced late-onset anxiety-like behavior and altered glutamate neurotransmission within the CeA, that parallels clinical studies implicating dysfunctional amygdala processing and the expression of affective symptomatology post-injury (Etkin and Wager, 2007; Koenigs and Grafman, 2009; Depue et al., 2014; Buchsbaum et al., 2015; Hrybouski et al., 2016; Stevens et al., 2016). Decreased BDNF/TrkB protein levels implicate one pathway by which dTBI can influence glutamate neurotransmission and thereby anxiety-like symptoms. Identification of common pathways for the development of TBI-induced and non-TBI-induced affective disorders are instrumental in treatment of symptoms, rehabilitation guidelines, and implementation of novel clinical approaches.

## Data Availability Statement

The datasets generated for this study are available on request to the corresponding author.

## Ethics Statement

The animal study was reviewed and approved by the Institutional Animal Care and Use Committee Protocol (13–460) at the University of Arizona College of Medicine-Phoenix.

## Author Contributions

TC conceived and designed the study. JB, TC, YH, SO, CB, and DG performed the experiments, analysis, figure preparation, and statistics. JB wrote the first draft. JB, PA, JL, HM, and TC interpreted the data. All authors edited the manuscript.

## Conflict of Interest

The authors declare that the research was conducted in the absence of any commercial or financial relationships that could be construed as a potential conflict of interest.

## Footnotes

1 https://imagej.nih.gov/ij/

2 http://imagej.net/AnalyzeSkeleton31

## Supplementary Material

FIGURE S1 Schematic of glutamate sensitive MEA. Ascorbic acid and other aromatic compounds are repelled from the MEA due to the electroplated 1,3-phenylenediamine (mPD) exclusion layer. Glutamate sensing (bottom pair) and sentinel (top pair) recording sites are coated with BSA and glutaraldehyde. Glutamate oxidase is added to signal sites only to convert glutamate to α-ketoglutarate and peroxide. Peroxide is oxidized on the platinum recording sites as a result of an applied potential of 0.7 V vs. Ag/AgCl reference electrode, thereby producing a current that is correlated with the concentration of glutamate present. Sentinel sites (top pair) are unable to record glutamate due to the lack of glutamate oxidase. Top left corner depicts an MEA. Schematic and MEA are not to scale.

FIGURE S2 Representative confirmation of MEA placement. Following amperometric experiments, brains were harvested and fixed for MEA placement. Brains were cut coronally at 40 μm. Here, a representative image of MEA placement into the anatomical ventral lateral hemisphere of the rat is shown. The black arrows indicate the outer track (BLA) and the inner track (CeA).

FIGURE S3 Skeletal Analysis Technique. Individual steps for skeleton analysis of microglia morphologies of Iba1 stained tissue. Original photomicrographs were subjected to a series of uniform ImageJ plugin protocols prior to conversion to binary images which were then skeletonized. An overlay of a resulting skeletonized image (in green) and original photomicrograph shows the relationship between skeleton and photomicrograph. All skeleton analysis was completed on full sized photomicrographs (40× magnification; scale bar = 10 μm).

FIGURE S4 Controls for applied solutions in amperometric recordings. Anesthetized *in vivo* amperometric recordings of glutamate neurotransmission were conducted in sham, 7 DPI, and 28 DPI rats in the BLA (A/P: −2.4 M/L ± 5.1 D/V −8.0) or CeA (A/P: −2.4 M/L ± 3.9 D/V −8.0). Local applications of 120 mM potassium chloride solution (KCl) were made to depolarize surrounding neurons. No differences in the volume locally applied was observed between sham and brain-injured rats in the **(A)** BLA [One-way ANOVA F(2,24) = 0.19; p = 0.83; *n* = 7*–*9] or **(D)** CeA [One-way ANOVA F(2,19) = 0.68; p = 0.52; *n* = 6*–*9]. Local applications of exogenous 100 μM were made to measure extracellular glutamate clearance. All applied peaks were amplitude matched prior to analysis in the **(B)** BLA [One-way ANOVA F(2,21) = 0.23; p = 0.79; *n* = 6*–*10] and **(E)** CeA[One-way ANOVA F(2,22) = 1.05; p = 0.37; *n* = 7*–*10] to control for Michaelis Menten kinetics. The maximum amplitude for evoked glutamate release in the BLA did not differ **(C)** [One-way ANOVA F(2,24) = 0.11; p = 0.90; *n* = 7*–*9], but was significantly decreased in the CeA at 28 DPI in comparison to sham **(F)** [One-way ANOVA F(2,19) = 4.89; *p* < 0.05; *n* = 6*–*9]. Bar graphs represent the mean ± SEM.

FIGURE S5 BLA and CeA microglia respond differently to dTBI over time. Microglial morphology changes as a function of time post-injury and region. **(A)** Microglial cell counts were different than sham after 1, 7, and 28 DPI in the BLA [Two-way ANOVA F(3,16) = 7.85; p < 0.05] **(B)**. The number of microglial process endpoints/cell was lower than sham after mFPI in the CeA [(3,16) = 5.152; p < 0.05]. **(C)** The summed process length/cell were lower than sham after mFPI in the CeA [F(3,16) = 3.94; p < 0.05]. Bar graphs represent mean ± SEM, *n* = 3/group. ^∗^*p* < 0.05 in comparison to sham. ^+^*p* < 0.05 in comparison to 1 DPI.

FIGURE S6 Representation of glutamatergic targets for protein quantification studies. Representation of a glutamatergic synapse and the proteins that regulate glutamate neurotransmission. Diagram modified from Thomas et al. (2012).

TABLE S1 Optimization parameters for antibodies and protein concentrations.
