# Corrigendum: Experimental Traumatic Brain Injury Induces Chronic Glutamatergic Dysfunction in Amygdala Circuitry Known to Regulate Anxiety-Like Behavior

**DOI:** 10.3389/fnins.2020.00212

**Published:** 2020-03-13

**Authors:** Joshua A. Beitchman, Daniel R. Griffiths, Yerin Hur, Sarah B. Ogle, Caitlin E. Bromberg, Helena W. Morrison, Jonathan Lifshitz, P. David Adelson, Theresa Currier Thomas

**Affiliations:** ^1^Barrow Neurological Institute at Phoenix Children's Hospital, Phoenix, AZ, United States; ^2^Department of Child Health, University of Arizona College of Medicine-Phoenix, Phoenix, AZ, United States; ^3^College of Graduate Studies, Midwestern University, Glendale, AZ, United States; ^4^Banner University Medical Center, Phoenix, AZ, United States; ^5^College of Nursing, University of Arizona, Tucson, AZ, United States; ^6^Phoenix VA Health Care System, Phoenix, AZ, United States

**Keywords:** diffuse traumatic brain injury, amperometry, glutamate neurotransmission, amygdala, chronic

In the original article, there was a mistake in the legend for Figure 2. The legends for Figure 2B and Figure 2C are switched. The correct legend appears below.

**“Figure 2**. TBI induces altered glutamate neurotransmission in the CeA. **(A)** Representative calibration of glutamate selective MEA. Arrows represent aliquots of solution of either 250 μM ascorbic acid (AA), 20 μM glutamate (Glu), or 8.8 μM H_2_O_2_. **(B)** Calculations for extracellular clearance of glutamate following local applications of 100 μM glutamate. **(C)** Calculations for total evoked glutamate release following local applications of 120 mM potassium chloride solution (KCl). **(D)** No significant differences were observed in evoked glutamate release in the BLA [Kruskal Wallis *H* = 4.63; *p* = 0.10; *n* = 8–11]. **(E)** No changes in glutamate clearance were observed when assessing the uptake rate [One-way ANOVA *F*(2,21) = 1.24; *p* = 0.31] or **(F)** *T*_80_ [One-way ANOVA *F*(2,21) = 0.14; *p* = 0.87; *n* = 6–10]. **(G)** Representative traces of KCl-evoked glutamate release. Baseline levels have been adjusted to show comparison amongst the three time points. Arrow represents 120 mM KCl administration. **(H)** 28 DPI rats had 50% less total evoked glutamate when compared to shams [One way ANOVA *F*(2,19) = 4.74 *p* < 0.05 Dunnett's *post hoc p* < 0.05, η^2^ = 0.33; *n* = 6–9] in the CeA. **(I)** Representative traces of extracellular glutamate clearance. Baselines levels have been adjusted to show comparison amongst the three time points. Arrow represents 100 μM glutamate administration. **(J)** 7 and 28 DPI rats had 40% and 58% slower uptake rate, respectively [One-way ANOVA *F*(2,22) = 7.88; *p* < 0.01, @ 7 DPI Dunnett's *post hoc p* < 0.05, @ 28 DPI Dunnett's *post hoc p* < 0.01, η^2^ = 0.41; *n* = 7–10]. **(K)** 28 DPI rats had a 43% increase in *T*_80_ [One-way ANOVA *F*(2,22) = 5.00; *p* < 0.05, Dunnett's *post hoc p* < 0.01, η^2^ = 0.33; *n* = 7–10]. **(L)** Uptake rate remained consistent between subsequent additions of glutamate to the CeA [RM Two-way ANOVA *F*(2,21) = 8.16; *p* < 0.01; *n* = 7–10]. Bar graphs represents mean ± SEM. ^*^p < 0.05 in comparison to sham.”

In addition, the references to Figure 2 in the text were also switched. In the methods section, the text referring to the Figure 2B should be Figure 2C. The text referring to Figure 2C should be Figure 2B.

A correction has been made to the Materials and Methods section, subsection Electrochemistry: KCl-Evoked Release of Glutamate Analysis Parameters, in paragraph 1.

“Once the electrochemical signal had reached baseline, 120 mM KCl was locally applied (BLA: 110 ± 8 nl; CeA: 105 ± 4 nl) to produce an evoked glutamate release. Additional ejections of KCl were completed at 2-min intervals and were volume matched at the time of administration. Criteria for analysis required that the peak with the largest amplitude was acquired from the first local application of KCl. This ensured that the data chosen were most representative of the maximum glutamate released within the surrounding neuronal tissue. Primary outcome measures were area under the curve as a proxy to investigate the total glutamate release capable of the recorded region. For a diagrammatic representation of these calculations, see Figure 2C.”

A correction has also been made to the Materials and Methods section, subsection Electrochemistry: Glutamate Clearance Analysis Parameters, in paragraph 1:

“Once the baseline was reached and maintained for at least 2 min (10–20 min), 100 μM glutamate was locally applied into the extracellular space (BLA: 73 ± 17 nl; CeA: 78 ± 15 nl). Exogenous glutamate was released at 30 s intervals and amplitude was matched at the time of administration. In analysis, three peaks were selected based on a predetermined amplitude range of 15 to 25 μM to ensure that data chosen were most representative of the glutamate clearance of similar volume and amplitude in accordance with Michaelis-Menton kinetics clearance parameters. The parameters for the 3 peaks were then averaged to create a single representative value per recorded region per rat. Primary outcome measures analyzed the uptake rate and the time taken for 80% of the maximum amplitude of glutamate to clear the extracellular space (*T*_80_). The uptake rate was calculated using the uptake rate constant (k_−1_) multiplied by the peak's maximum amplitude, thus controlling for any variation between the amount of applied glutamate between peaks. For a diagrammatic representation of these calculations, see Figure 2B.”

The authors apologize for this error and state that this does not change the scientific conclusions of the article in any way. The original article has been updated.

