# Supplementary figures and images for: Experimental Traumatic Brain Injury Induces Chronic Glutamatergic Dysfunction in Amygdala Circuitry Known to Regulate Anxiety-Like Behavior
Source: Front Neurosci. 2020 Jan 21;13:1434. doi: 10.3389/fnins.2019.01434 (PMC6985437; doi:10.3389/fnins.2019.01434)

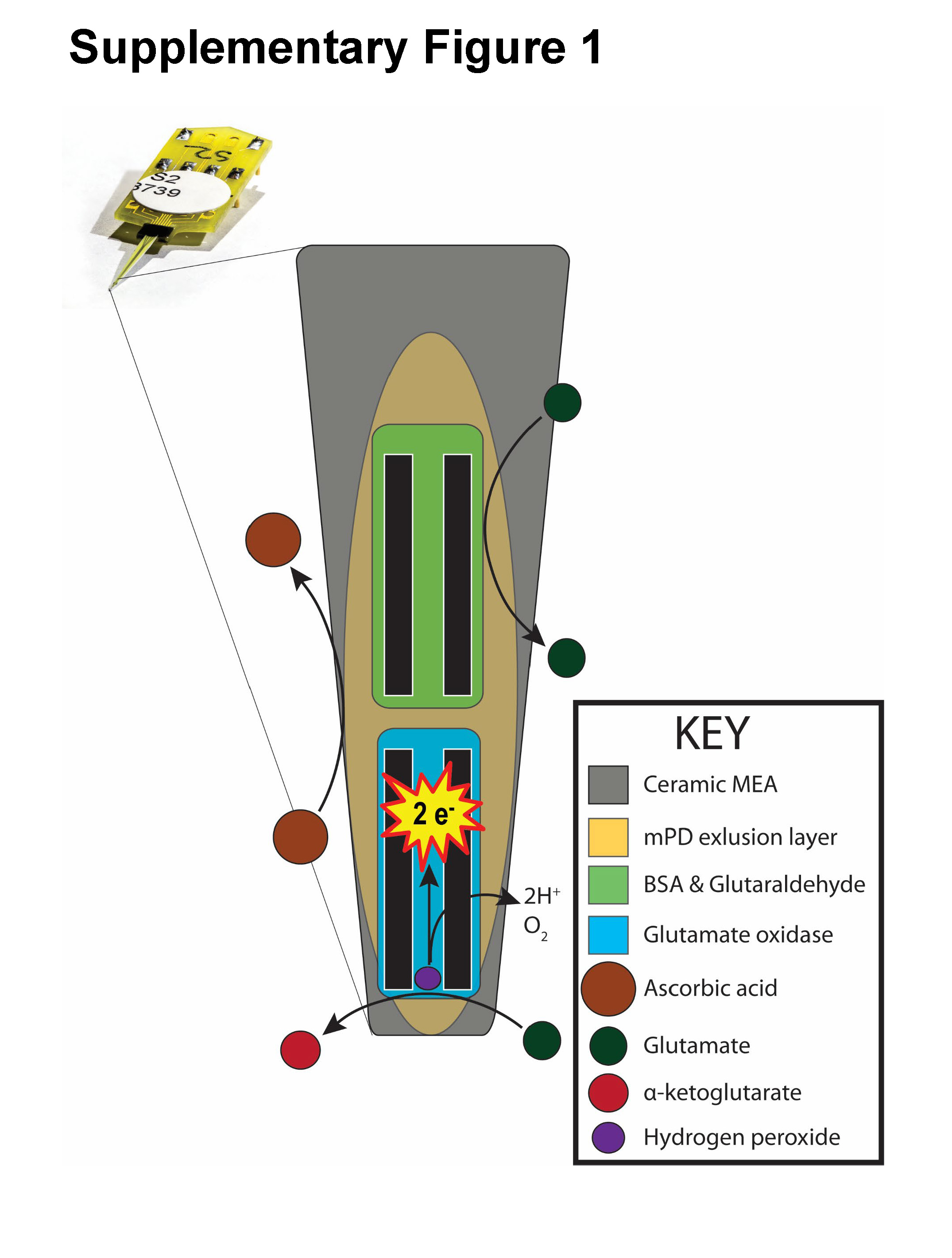

Supplement: FIGURE S1 — Schematic of glutamate sensitive MEA. Ascorbic acid and other aromatic compounds are repelled from the MEA due to the electroplated 1,3-phenylenediamine (mPD) exclusion layer. Glutamate sensing (bottom pair) and sentinel (top pair) recording sites are coated with BSA and glutaraldehyde. Glutamate oxidase is added to signal sites only to convert glutamate to α-ketoglutarate and peroxide. Peroxide is oxidized on the platinum recording sites as a result of an applied potential of 0.7 V vs. Ag/AgCl reference electrode, thereby producing a current that is correlated with the concentration of glutamate present. Sentinel sites (top pair) are unable to record glutamate due to the lack of glutamate oxidase. Top left corner depicts an MEA. Schematic and MEA are not to scale. [file Image_1.JPEG]

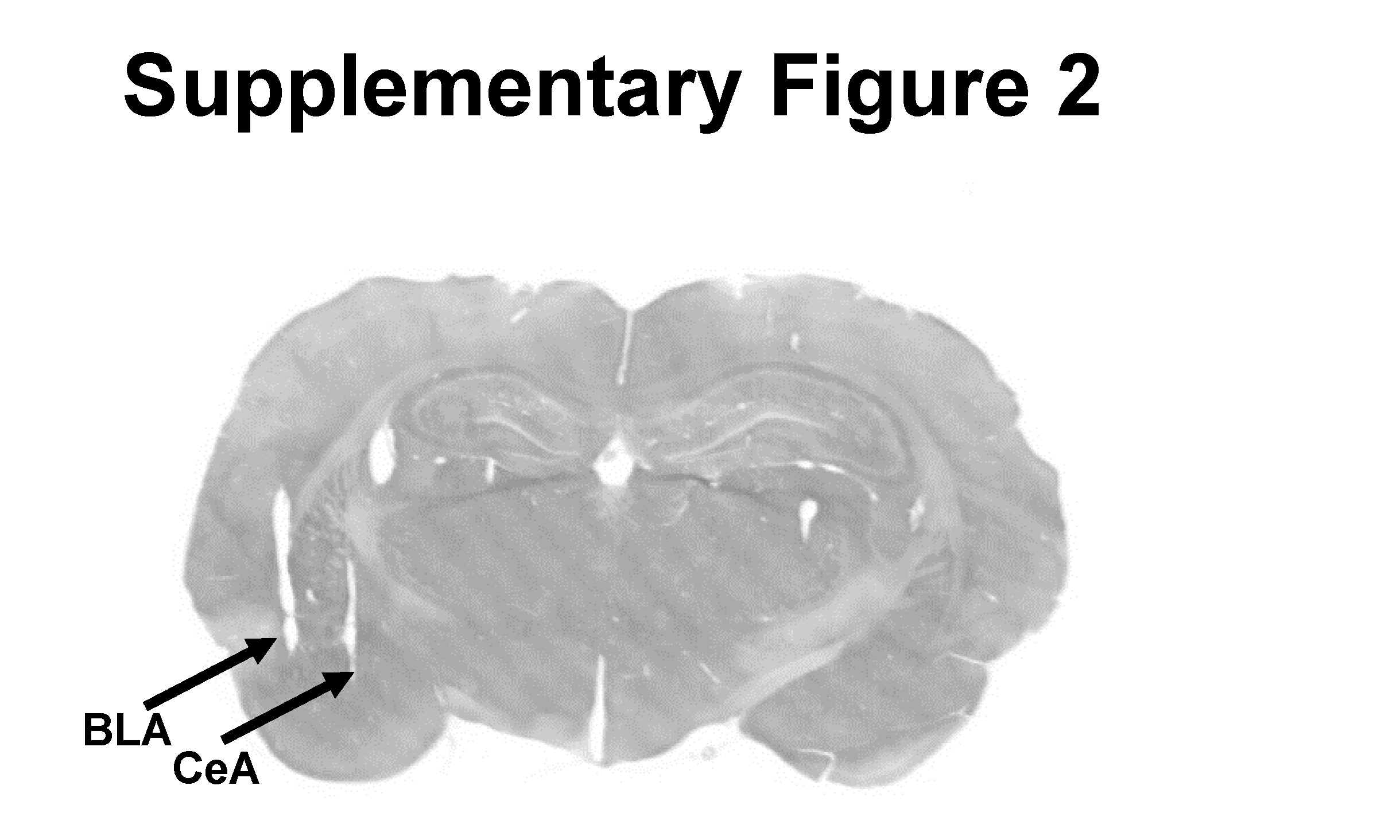

Supplement: FIGURE S2 — Representative confirmation of MEA placement. Following amperometric experiments, brains were harvested and fixed for MEA placement. Brains were cut coronally at 40 μm. Here, a representative image of MEA placement into the anatomical ventral lateral hemisphere of the rat is shown. The black arrows indicate the outer track (BLA) and the inner track (CeA). [file Image_2.TIFF]

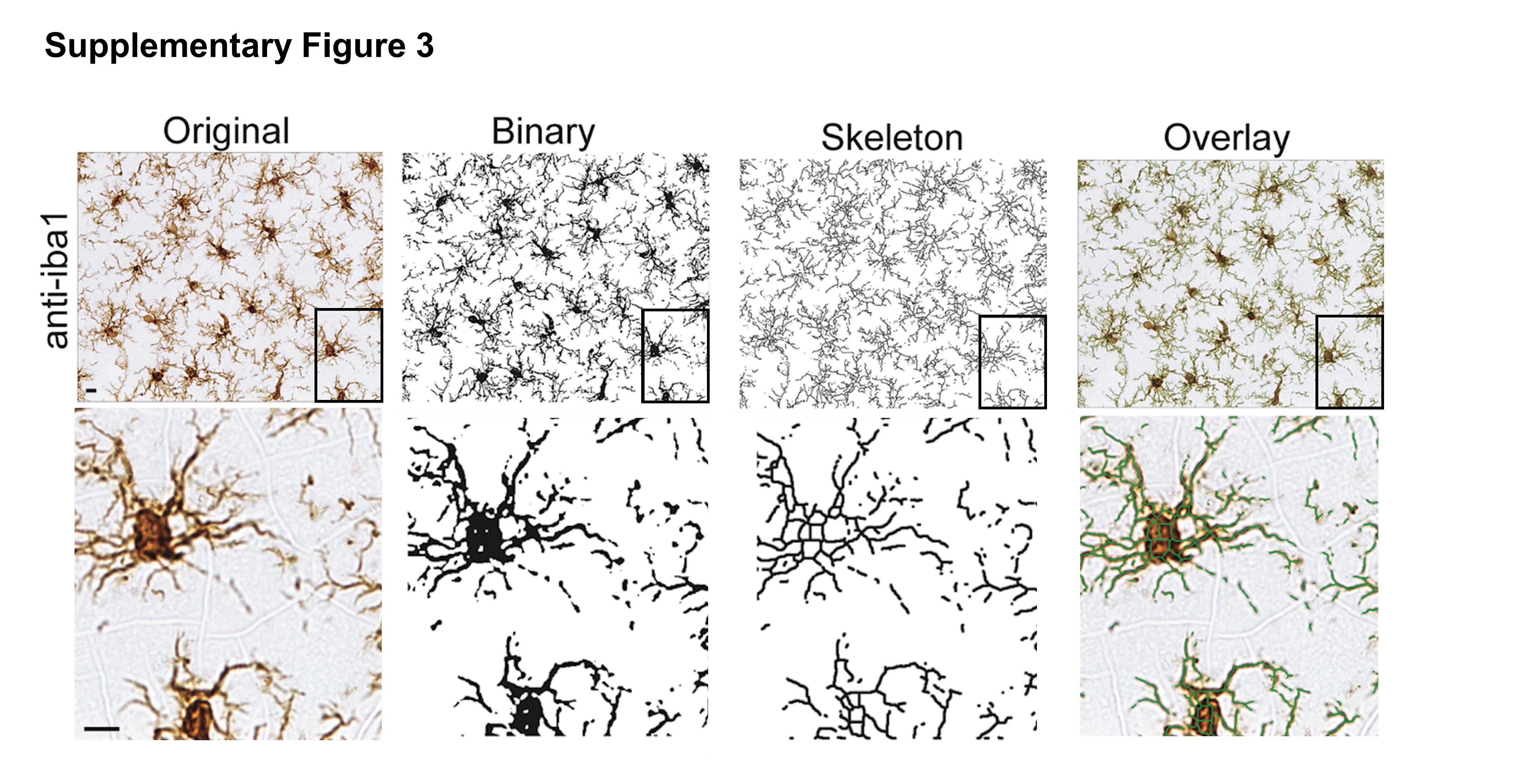

Supplement: FIGURE S3 — Skeletal Analysis Technique. Individual steps for skeleton analysis of microglia morphologies of Iba1 stained tissue. Original photomicrographs were subjected to a series of uniform ImageJ plugin protocols prior to conversion to binary images which were then skeletonized. An overlay of a resulting skeletonized image (in green) and original photomicrograph shows the relationship between skeleton and photomicrograph. All skeleton analysis was completed on full sized photomicrographs (40× magnification; scale bar = 10 μm). [file Image_3.JPEG]

# Supplementary Figure 4

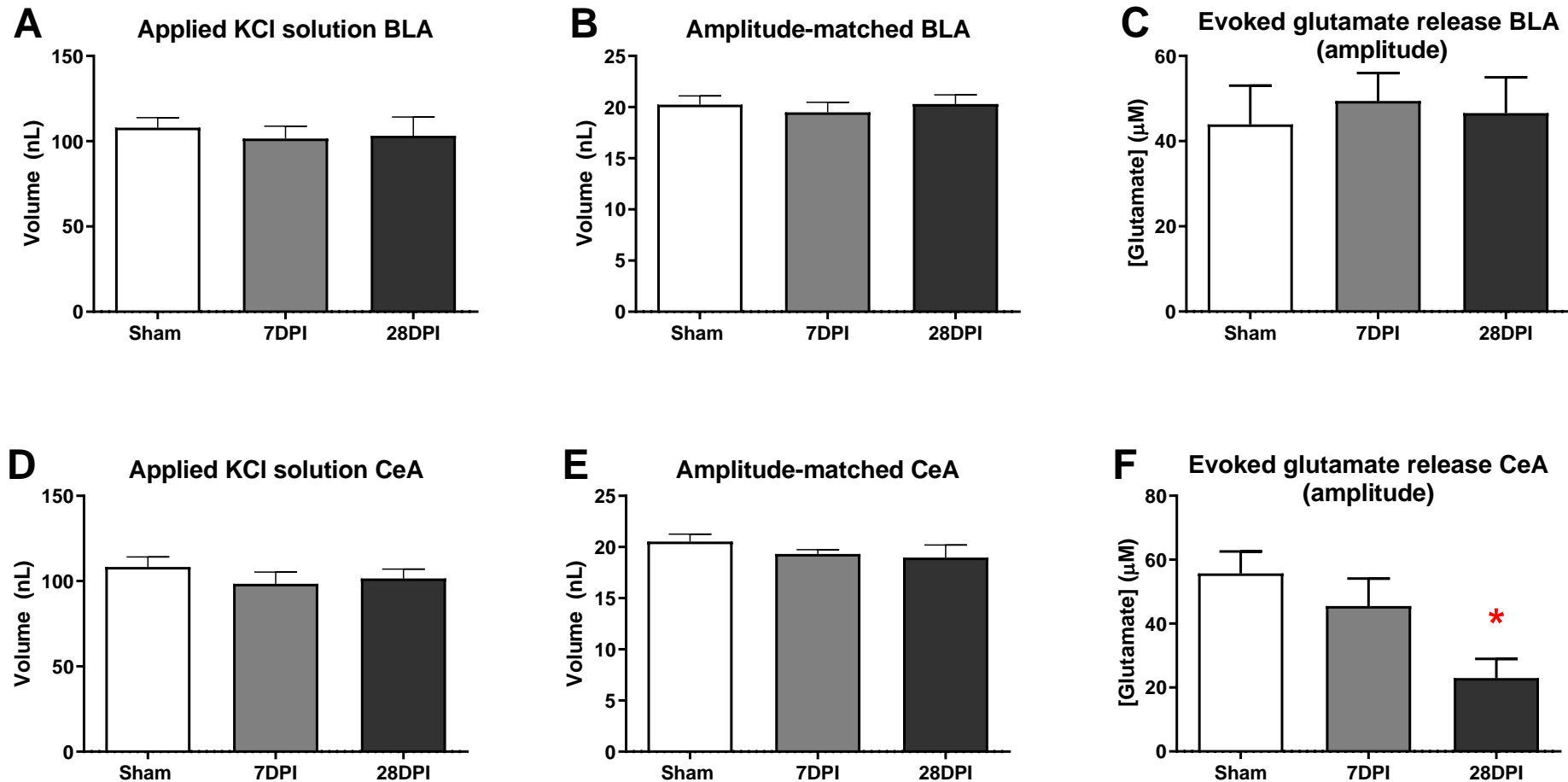

Supplement: FIGURE S4 — Controls for applied solutions in amperometric recordings. Anesthetized in vivo amperometric recordings of glutamate neurotransmission were conducted in sham, 7 DPI, and 28 DPI rats in the BLA (A/P: −2.4 M/L ± 5.1 D/V −8.0) or CeA (A/P: −2.4 M/L ± 3.9 D/V −8.0). Local applications of 120 mM potassium chloride solution (KCl) were made to depolarize surrounding neurons. No differences in the volume locally applied was observed between sham and brain-injured rats in the (A) BLA [One-way ANOVA F(2,24) = 0.19; p = 0.83; n = 7–9] or (D) CeA [One-way ANOVA F(2,19) = 0.68; p = 0.52; n = 6–9]. Local applications of exogenous 100 μM were made to measure extracellular glutamate clearance. All applied peaks were amplitude matched prior to analysis in the (B) BLA [One-way ANOVA F(2,21) = 0.23; p = 0.79; n = 6–10] and (E) CeA[One-way ANOVA F(2,22) = 1.05; p = 0.37; n = 7–10] to control for Michaelis Menten kinetics. The maximum amplitude for evoked glutamate release in the BLA did not differ (C) [One-way ANOVA F(2,24) = 0.11; p = 0.90; n = 7–9], but was significantly decreased in the CeA at 28 DPI in comparison to sham (F) [One-way ANOVA F(2,19) = 4.89; p < 0.05; n = 6–9]. Bar graphs represent the mean ± SEM. [file Image_4.pdf]

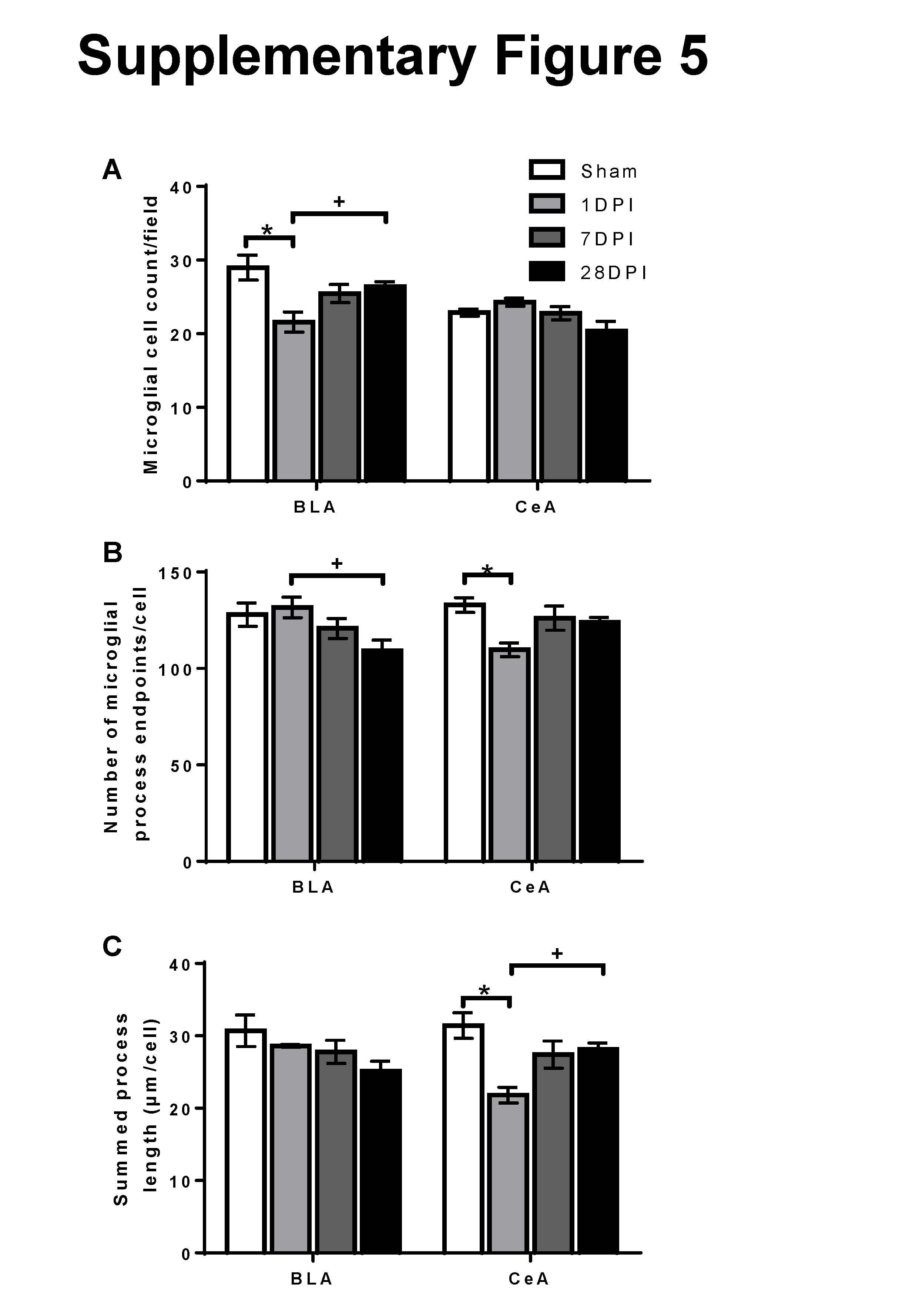

Supplement: FIGURE S5 — BLA and CeA microglia respond differently to dTBI over time. Microglial morphology changes as a function of time post-injury and region. (A) Microglial cell counts were different than sham after 1, 7, and 28 DPI in the BLA [Two-way ANOVA F(3,16) = 7.85; p < 0.05] (B). The number of microglial process endpoints/cell was lower than sham after mFPI in the CeA [(3,16) = 5.152; p < 0.05]. (C) The summed process length/cell were lower than sham after mFPI in the CeA [F(3,16) = 3.94; p < 0.05]. Bar graphs represent mean ± SEM, n = 3/group. ∗p < 0.05 in comparison to sham. +p < 0.05 in comparison to 1 DPI. [file Image_5.TIFF]

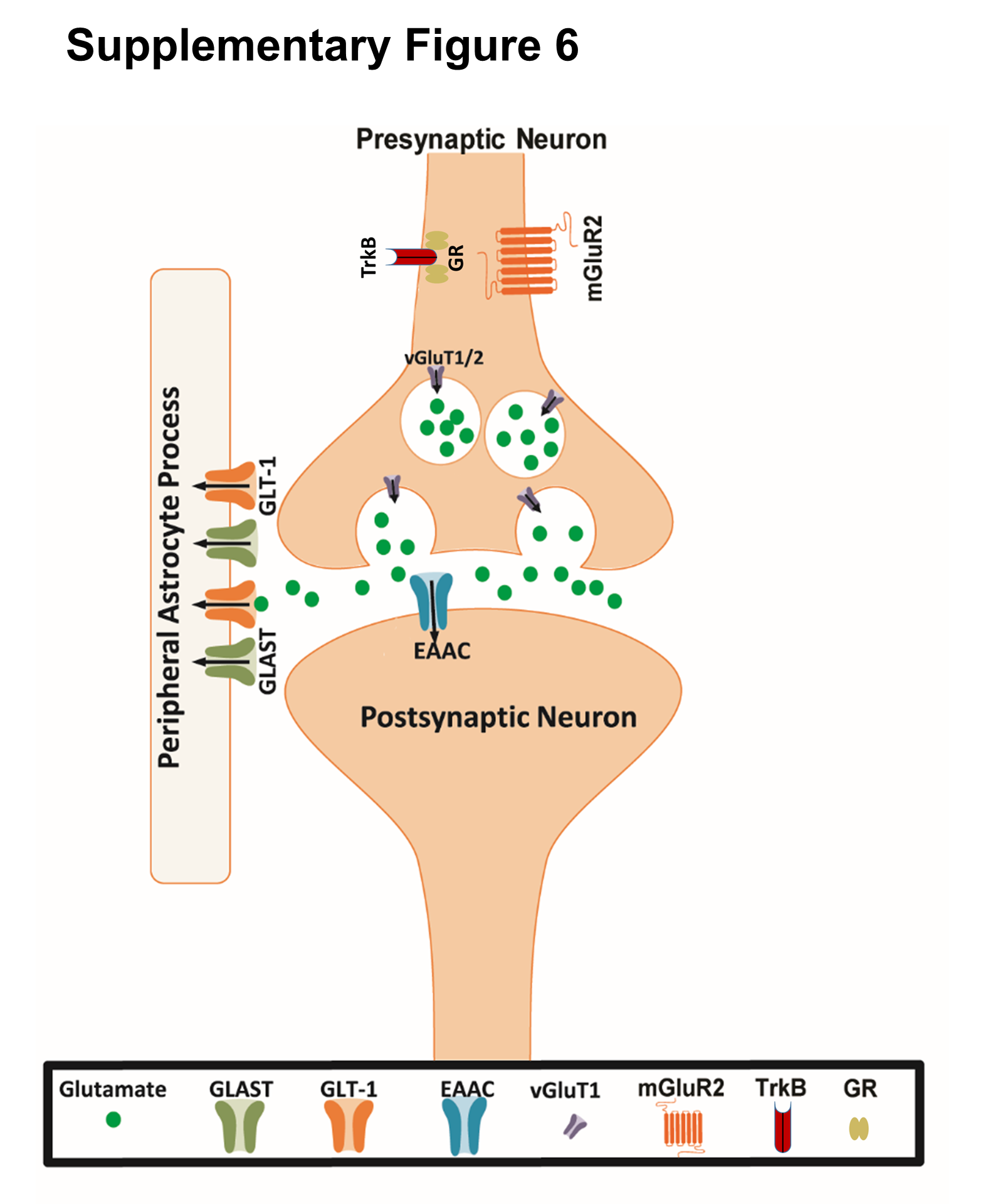

Supplement: FIGURE S6 — Representation of glutamatergic targets for protein quantification studies. Representation of a glutamatergic synapse and the proteins that regulate glutamate neurotransmission. Diagram modified from Thomas et al. (2012). [file Image_6.TIF]
