# Supplementary material for: Experimental Traumatic Brain Injury Induces Chronic Glutamatergic Dysfunction in Amygdala Circuitry Known to Regulate Anxiety-Like Behavior
Source: Front Neurosci. 2020 Jan 21;13:1434. doi: 10.3389/fnins.2019.01434 (PMC6985437; doi:10.3389/fnins.2019.01434)
Supplement: TABLE S1 — Optimization parameters for antibodies and protein concentrations. [file Table_1.pdf]

Antibody	Species	Ig company, catalog #	Denaturing Temp (celcius)	[Protein] µg/µl	[Ig]	Biological control
BDNF	Mouse	Abcam ab108319	37Cx30min	0.5 µg/µl	1:50	GAPDH
GLUR/NR3C1	Rb	Neo A2164	37Cx30min	0.5 µg/µl	1:25	GAPDH
TrkB	Rb	Abcam ab18987	37Cx30 min	0.5 µg/µl	1:100	GAPDH
GLT-1 (EAAT2)	Rb	Abcam ab205248	37Cx30 min	0.1 µg/µl	1:25	GAPDH
Glast (EAAT1)	Rb	Abcam ab181036	37Cx30 min	0.5 µg/µl	1:25	GAPDH
mGluR2	Rb	Abcam ab150387	37Cx30 min	0.1 µg/µl	1:100	GAPDH
Gapdh	mouse	Abcam ab8245	37Cx30 min	0.5 µg/µl	1:25	
